# Deep Parallel Characterization of AAV Tropism and AAV-Mediated Transcriptional Changes *via* Single-Cell RNA Sequencing

**DOI:** 10.3389/fimmu.2021.730825

**Published:** 2021-10-21

**Authors:** David Brown, Michael Altermatt, Tatyana Dobreva, Sisi Chen, Alexander Wang, Matt Thomson, Viviana Gradinaru

**Affiliations:** ^1^ Division of Biology and Biological Engineering, California Institute of Technology, Pasadena, CA, United States; ^2^ Andrew and Peggy Cherng Department of Medical Engineering, California Institute of Technology, Pasadena, CA, United States; ^3^ Division of Engineering and Applied Science, California Institute of Technology, Pasadena, CA, United States

**Keywords:** gene therapy, next generation sequencing, computational biology, molecular biology, neuroscience

## Abstract

Engineered variants of recombinant adeno-associated viruses (rAAVs) are being developed rapidly to meet the need for gene-therapy delivery vehicles with particular cell-type and tissue tropisms. While high-throughput AAV engineering and selection methods have generated numerous variants, subsequent tropism and response characterization have remained low throughput and lack resolution across the many relevant cell and tissue types. To fully leverage the output of these large screening paradigms across multiple targets, we have developed an experimental and computational single-cell RNA sequencing (scRNA-seq) pipeline for *in vivo* characterization of barcoded rAAV pools at high resolution. Using this platform, we have both corroborated previously reported viral tropisms and discovered unidentified AAV capsid targeting biases. As expected, we observed that the tropism profile of AAV.CAP-B10 in mice was shifted toward neurons and away from astrocytes when compared with AAV-PHP.eB. Transcriptomic analysis revealed that this neuronal bias is due mainly to increased targeting efficiency for glutamatergic neurons, which we confirmed by RNA fluorescence *in situ* hybridization. We further uncovered cell subtype tropisms of AAV variants in vascular and glial cells, such as low transduction of pericytes and Myoc+ astrocytes. Additionally, we have observed cell-type-specific transitory responses to systemic AAV-PHP.eB administration, such as upregulation of genes involved in p53 signaling in endothelial cells three days post-injection, which return to control levels by day twenty-five. The presented experimental and computational approaches for parallel characterization of AAV tropism will facilitate the advancement of safe and precise gene delivery vehicles, and showcase the power of understanding responses to gene therapies at the single-cell level.

## 1 Introduction

Recombinant AAVs (rAAVs) have become the preferred gene delivery vehicles for many clinical and research applications (1, 2) owing to their broad viral tropism, ability to transduce dividing and non-dividing cells, low immunogenicity, and stable persistence as episomal DNA ensuring long-term transgene expression (3–8). However, current systemic gene therapies using AAVs have a relatively low therapeutic index (9). High doses are necessary to achieve sufficient transgene expression in target cell populations, which can lead to severe adverse effects from off-target expression (10–12). Increased target specificity of rAAVs would reduce both the necessary viral dose and off-target effects; thus, there is an urgent need for AAV gene delivery vectors that are optimized for cell-type-specific delivery (13). Lower viral doses would also alleviate demands on vector manufacturing and minimize the chances of undesirable immunological responses (14–16). Capsid-specific T-cell activation was reported to be dose-dependent *in vitro* (17, 18) and in humans (19, 20). Shaping the tropism of existing AAVs to the needs of a specific disease has the potential to reduce activation of the immune system by detargeting cell types, such as dendritic cells, that have an increased ability to activate T-cells (21–26).

Several studies have demonstrated that the transduction efficiency and specificity of natural AAVs can be improved by engineering their capsids using rational design (27–31) or directed evolution (32–47). These engineering methods yield diverse candidates that require thorough, preferably high-throughput, *in vivo* vector characterization to identify optimal candidates for a particular clinical or research application. Toward this end, conventional immunohistochemistry (IHC) and various *in situ* hybridization (ISH) techniques are commonly employed to profile viral tropism by labeling proteins expressed by the viral transgene or viral nucleic acids, respectively (10, 32, 34, 45, 48–59).

Although these histological approaches preserve spatial information, current technical challenges limit their application to profiling the viral tropism of just one or two AAV variants across a few gene markers, thus falling short of efficiently characterizing multiple AAVs across many complex cell types characteristic of tissues in the central nervous system (CNS). The reliance on known marker genes also prevents the unbiased discovery of tropisms since such marker genes need to be chosen *a priori*. Choosing marker genes is particularly challenging for supporting cell types, such as pericytes in the CNS microvasculature and oligodendrocytes, which often have less established cell type identification strategies (60, 61). The advent of single-cell RNA sequencing (scRNA-seq) has enabled comprehensive transcriptomic analysis of entire cell-type hierarchies, and brought new appreciation to the role of cell subtypes in disease (62–66). However, experimental and computational challenges, such as the sparsity of RNA capture and detection, strong batch effects between samples, and the presence of ambient RNA in droplets, reduce the statistical confidence of claims about individual gene expression (67–69). Computational methods have been developed to address some of these challenges, such as identifying contaminating RNA (68), accounting for or removing batch effects (70–72), and distinguishing intact cells from empty droplets (69, 73, 74). However, strategies for simultaneously processing transcripts from multiple delivery vehicles and overcoming the computational challenges of confidently detecting individual transcripts have not yet been developed for probing the tropism of AAVs in complex, heterogeneous cell populations.

Collecting the entire transcriptome of injected and non-injected animals also offers an opportunity to study the effects of AAV transduction on the host cell transcriptome. A similar investigation has been conducted with G-deleted rabies virus (75). This study demonstrated that virus infection led to the downregulation of genes involved in metabolic processes and neurotransmission in host cells, whereas genes related to cytokine signaling and the adaptive immune system were upregulated. At present, no such detailed examination of transcriptome changes upon systemic AAV injection has been conducted. High-throughput single-cell transcriptomic analysis could provide further insight into the ramifications of AAV capsid and transgene modifications with regard to innate (76–80) and adaptive immune recognition (20, 81–84). Innate and adaptive immune responses to AAV gene delivery vectors and transgene products constitute substantial hurdles to their clinical development (85, 86). The study of brain immune response to viral gene therapy has been limited to antibody staining and observation of brain tissue slices post direct injection. In particular, prior studies have shown that intracerebral injection of rAAV vectors in rat brains does not induce leukocytic infiltration or gliosis (87, 88); however, innate inflammatory responses were observed (89). Results reported by these methods are rooted in single-marker staining and thus prevent the discovery of unexpected cell-type-specific responses. A comprehensive understanding of the processes underlying viral vector or transgene-mediated responses is critical for further optimizing AAV gene delivery vectors and treatment modalities that mitigate such immune responses.

**Figure 1:**
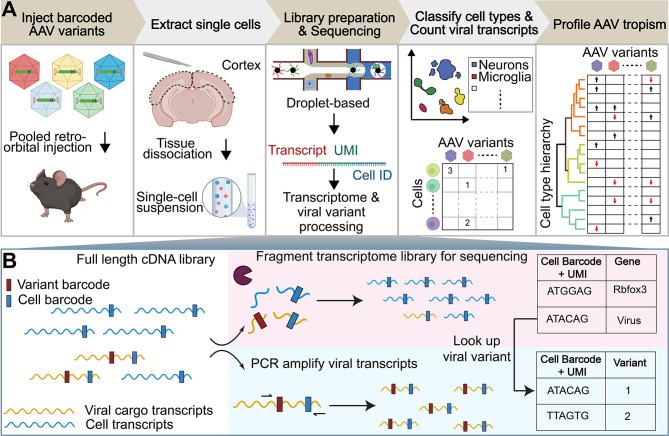
Workflow of AAV tropism characterization by scRNA-seq. **(A)** (I) Injection of a single AAV variant or multiple barcoded AAV variants into the retro-orbital sinus. (II) After 3–4 weeks post-injection, the brain region of interest is extracted and the tissue is dissociated into a single-cell suspension. (III) The droplet-based 10x Genomics Chromium system is used to isolate cells and build transcriptomic libraries [see **(B)**]. (IV) Cells are assigned a cell-type annotation and a viral transcript count. (V) AAV tropism profiling across numerous cell types. **(B)** The full length cDNA library is fragmented for sequencing as part of the single-cell sequencing protocol (top). To enable viral tropism characterization of multiple rAAVs in parallel, an aliquot of the intact cDNA library undergoes further PCR amplification of viral transcripts (bottom). During cDNA amplification, Illumina sequencing primer targets are added to the viral transcripts such that the sequence in between the Illumina primer targets contains the AAV capsid barcode sequence. Viral cargo in the cell transcriptome is converted to variant barcodes by matching the corresponding cell barcode + UMI in the amplified viral transcript library (right).

Here, we introduce an experimental and bioinformatics workflow capable of profiling the viral tropism and response of multiple barcoded AAV variants in a single animal across numerous complex cell types by taking advantage of the transcriptomic resolution of scRNA-seq techniques (**Figure 1A**). For this proof-of-concept study, we profile the tropism of previously-characterized AAV variants that emerged from directed evolution with the CREATE (AAV-PHP.B, AAV-PHP.eB) (32, 34) or M-CREATE (AAV-PHP.C1, AAV-PHP.C2, AAV-PHP.V1, AAV.CAP-B10) (45, 90) platforms. We selected the AAV variants based on their unique CNS tropism following intravenous injection. AAV-PHP.B and AAV-PHP.eB are known to exhibit overall increased targeting of the CNS compared with AAV9 and preferential targeting of neurons and astrocytes. Despite its sequence similarity to AAV-PHP.B, the tropism of AAV-PHP.V1 is known to be biased toward transducing brain vascular cells. AAV-PHP.C1 and AAV-PHP.C2 have both demonstrated enhanced blood–brain barrier (BBB) crossing relative to AAV9 across two mouse strains (C57BL/6J and BALB/cJ). Finally, AAV.CAP-B10 is a recently-developed variant with a bias toward neurons compared to AAV-PHP.eB (90).

In the initial validation experiment, we quantify the transduction biases of AAV-PHP.eB and AAV.CAP-B10 across major cell types using scRNA-seq, and demonstrate results which correlate well with both published results and conventional IHC-based quantification. We then demonstrate the power of the transcriptomic approach by going beyond the major cell types to reveal significant differences in cell-subtype transduction specificity. Compared with AAV.CAP-B10, AAV-PHP.eB displays biased targeting of inhibitory neurons, and both variants transduce Sst+ or Pvalb+ inhibitory neurons more efficiently than Vip+ inhibitory neurons. We validate these results with fluorescent *in situ* hybridization – hybridization chain reaction (FISH-HCR). We then develop and validate a barcoding strategy to investigate the tropism of AAV-PHP.V1 relative to AAV-PHP.eB in non-neuronal cells and reveal that pericytes, a subclass of vascular cells, evade transduction by this and other variants. We further use scRNA-seq to profile cell-type-specific responses to AAV-PHP.eB at 3 and 25 days post-injection (DPI), finding numerous genes implicated in the p53 pathway in endothelial cells to be upregulated at 3 DPI and returning back to control levels at 25 DPI. Finally, we showcase the capabilities of parallel characterization by verifying the preceding findings in a single animal with seven co-injected AAV variants and revealing their respective cell-type biases.

## 2 Results

### 2.1 Multiplexed Single-Cell RNA Sequencing-Based AAV Profiling Pipeline

To address the current bottleneck in AAV tropism profiling, we devised an experimental and computational workflow (**Figure 1A**) that exploits the transcriptomic resolution of scRNA-seq to profile the tropism of multiple AAV variants across complex cell-type hierarchies. In this workflow, single or multiple barcoded rAAVs are injected into the retro-orbital sinus of mice followed by tissue dissociation, single-cell library construction using the 10X Genomics Chromium system, and sequencing with multiplexed Illumina next-generation sequencing (NGS) (69). The standard mRNA library construction procedure includes an enzymatic fragmentation step that truncates the cDNA amplicon such that its final size falls within the bounds of NGS platforms (**Figure 1B**). These cDNA fragments are only approximately 450 bp in length and, due to the stochastic nature of the fragmentation, sequencing from their 5’ end does not consistently capture any particular region. The fragment length limit and heterogeneity pose a problem for parallelizing AAV tropism profiling, which requires reliable recovery of regions of the transgene that identify the originating AAV capsid. For example, posttranscriptional regulatory elements, such as the 600 bp Woodchuck hepatitis virus posttranscriptional regulatory element (WPRE), are commonly placed at the 3’ end of viral transgenes to modulate transgene expression. The insertion of such elements pushes any uniquely identifying cargo outside the 450 bp capture range, making them indistinguishable based on the cDNA library alone (**Supplemental Figure 1A**). An alternative strategy of adding barcodes in the 3’ polyadenylation site also places the barcode too distant for a 5’ sequencing read, and reading from the 3’ end would require sequencing through the homopolymeric polyA tail, which is believed to be unreliable in NGS platforms (91, 92).

**Figure 2:**
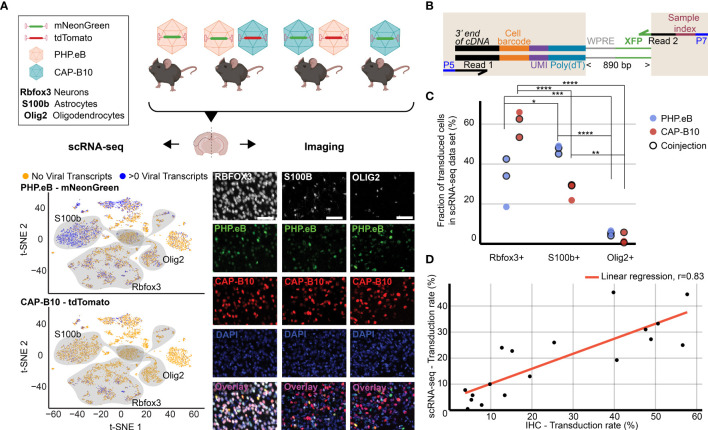
Comparison of viral tropism profiling with traditional IHC and scRNA-seq. **(A)** Overview of the experiment. Four animals were injected with 1.5 × 10_11_ viral genomes (vg) packaged in AAV-PHP.eB and/or AAV.CAP-B10. The bottom panels show a representative dataset collected from an animal that was co-injected with AAV-PHP.eB and AAV.CAP-B10. The left side displays the scRNA-seq dataset in the lower dimensional t-SNE space, with cells colored according to presence of viral transcripts. The shaded areas indicate clusters with high expression of the corresponding gene marker. The right side shows representative confocal images of cortical tissue labeled with IHC. Scale bar, 50 µm. **(B)** Viral transcript recovery strategy. The shaded areas highlight sequences added during library construction. **(C)** The fraction of the total number of transduced cells labeled as expressing the corresponding marker gene. For each AAV variant, the results of a two-way ANOVA with correction for multiple comparisons using Sidak’s test are reported with adjusted P-values (****P ≤ 0.0001, ***P ≤ 0.001, and **P ≤ 0.01, and *P ≤ 0.05 are shown; P > 0.05 is not shown). **(D)** Comparison of transduction rates based on quantification *via* scRNA-seq or IHC. Transduction rate in IHC was calculated as (number of transduced cells in the group)/(total number of cells in the group). Transduction rate in scRNA-seq is based on an estimate of the fraaction of cells containing transcripts above background (see *Methods*). Each dot represents the transduction rate of neurons/Rbfox3+, astrocytes/S100b+, or oligodendrocytes/Olig2+ by AAV-PHP.eB or AAV.CAP-B10 in one animal. Histology data are averages across three brain slices per gene marker and animal. r indicates the Pearson correlation coefficient.

We circumvented these limitations in viral cargo identification by taking an aliquot of the intact cDNA library and adding standard Illumina sequencing primer recognition sites to the viral transcripts using PCR amplification such that the identifying region is within the two Illumina primer target sequences (e.g. **Figure 2B**). The cell transcriptome aliquots undergoing the standard library construction protocol and the amplified viral transcripts are then sequenced as separate NGS libraries. We sequence shorter viral transcripts in the same flow cell as the cell transcriptomes and longer viral transcripts on the Illumina MiSeq, which we found to be successful at sequencing cDNAs up to 890 bp long. The sequencing data undergoes a comprehensive data processing pipeline (see *Methods*). Using a custom genome reference, reads from the cell transcriptome that align to the viral cargo plasmid sequences are counted as part of the standard 10X Cell Ranger count pipeline (see *Methods* and **Supplemental Figure 1C**). In parallel, reads from the amplified viral transcripts are used to count the abundance of each viral barcode associated with each cell barcode and unique molecular identifier (UMI). The most abundant viral barcode for each cell barcode and UMI is assumed to be the correct viral barcode, and is used to construct a variant lookup table. This lookup table approach identifies an originating capsid in 65.7 ± 2.3% of viral transcripts detected in the cell transcriptome aliquots (**Supplemental Table 4**).

For determining viral cell-type tropism, we developed a method to estimate the fraction of cells within a cell type that express viral transcripts. Viral RNA expression levels depend on both the multiplicity of infection and the transcription rate of the delivered cargo. Thus, directly using viral RNA counts to determine tropism is confounded by differences in transcription rate between cell types, limiting comparison with imaging-based tropism quantification methods. As evidence of this, we detected that viral RNA expression levels can vary by cell type but are not perfectly rank correlated with the percent of cells detected as expressing that transcript (**Supplemental Figure 2B**). An additional confound arises from the ambient RNA from cellular debris co-encapsulated with cell-containing droplets, which can lead to false positives, i.e., detecting viral RNA in droplets containing a cell that was not expressing viral RNA. For example, we detected low levels of viral transcripts in large percentages of cells, even in cell types suspected to evade transduction, such as immune cells (**Supplemental Figure 2A**). To reduce the effect of both variability in expression and ambient RNA, we developed an empirical method to estimate the percentage of cells expressing transcripts above the noise, wherein the distribution of viral transcript counts in a set of cells of interest is compared to a background distribution of cell-free (empty) droplets (see *Methods*, **Supplemental Figure 2C**). In simulation, this method accurately recovers the estimated number of cells expressing transcripts above background across a wide range of parameterizations of negative binomial distributions (see *Methods*, **Supplemental Figure 2D**). Importantly, this method can yield a different ranking of viral tropism as compared to mean transcript expression rate (**Supplemental Figure 2E**).

To address several additional technical problems in default single-cell pipelines, we developed a simultaneous quality control (QC) and droplet identification pipeline. Our viral transduction rate estimation method described above relies on having an empirical background distribution of viral transcript counts in empty droplets to compare against the cell type of interest. However, the default cell *vs.* empty droplet identification method provided by the 10X Cell Ranger software, which is based on the EmptyDrops method (73), yielded unexpectedly high numbers of cells and clusters with no recognizable marker genes, suggesting they may consist of empty droplets of ambient RNA or cellular debris (**Supplemental Figures 3A, B**). Additionally, we sought to remove droplets containing multiple cells (multiplets) from our data due to the risk of falsely attributing viral tropism of one cell type to another. However, using Scrublet (93), an established method for identifying droplets containing multiplets, failed to identify multiplets in some of our samples and only identified small proportions of clusters positive for known non-overlapping marker genes, such as Cldn5 and Cx3cr1 (**Supplemental Figure 3C**). To address both the empty droplet and multiplet detection issues, we built a droplet classification pipeline based on scANVI, a framework for classifying single-cell data *via* neural-network-based generative models (94). Using clusters with a high percentage of predicted multiplets from Scrublet as training examples of multiplets, and clusters positive for known neuronal and non-neuronal marker genes as training examples of neurons and non-neuronal cells, we trained a predictive model to classify each droplet as a neuron, non-neuron, multiplet, or empty droplet (see *Methods*, **Supplemental Figure 4A**). This model performed with 97.4% accuracy on 10% of cells held out for testing, and yielded a database of 334,151 cortical cells (**Supplemental Figure 4B**). Inspection of the cells classified as empty droplets reveals that these droplets have lower transcript counts and higher mitochondrial gene ratios, consistent with other single-cell quality control pipelines (**Supplemental Figure 4D**). Critically, we discovered that non-neuronal clusters contained significantly more cells that had been previously removed by the Cell Ranger filtering method as compared to neuronal clusters (P = 0.025, 2-sided student t-test). In some clusters within cell subtypes, such as mature oligodendrocytes and endothelial cells, we identified up to 67% more cells than what were recovered *via* Cell Ranger.

Using our combined experimental and computational pipeline for viral transcript recovery and droplet identification, we can recover a lower bound on the expected number of cells expressing each unique viral cargo within groups of cells in heterogeneous samples.

### 2.2 Single-Cell RNA Sequencing Recapitulates AAV Capsid Cell-Type-Specific Tropisms

As a first step, we validated our method by comparing the quantification of AAV transduction of major cell types *via* scRNA-seq to conventional IHC. For this purpose, we characterized the tropism of two previously reported AAV variants, AAV-PHP.eB (32) and AAV.CAP-B10 (90) (**Figure 2A**). In total, four animals received single or dual retro-orbital injections of AAV-PHP.eB and/or AAV.CAP-B10 with 1.5 × 10^11^ viral genomes (vg) per variant. Co-injection of both variants served to test the ability of our approach to parallelize tropism profiling. By having each variant package a distinct fluorophore, tropism could be simultaneously assessed *via* multi-channel fluorescence and mRNA expression of the distinct transgene. After 3–4 weeks of expression, we harvested the brains and used one hemisphere for IHC and one hemisphere for scRNA-seq. To recover viral transcripts, we chose primers such that enough of the XFP sequence was contained within the Illumina primer target sequences to differentiate the two variants (**Figure 2B**, **Supplemental Table 1**). For this comparison, we focused on the transduction rate for neurons (Rbfox3), astrocytes (S100b), and oligodendrocytes (Olig2). For IHC, a cell was classified as positive for the marker gene on the basis of antibody staining, and was classified as transduced on the basis of expression of the delivered fluorophore. For scRNA-seq, all cells that passed our QC pipeline were projected into a joint scVI latent space and clustered. To most closely match our imaging quantification, we considered all clusters that were determined to be positive for the respective marker gene as belonging to the corresponding cell type (see *Methods*). All clusters of the same marker gene were grouped together, and the transduction rate of the combined group of cells was determined using our viral transduction rate estimation method.

Our analysis of the scRNA-seq data demonstrates that the viral tropism biases across the three canonical marker genes are consistent with previous reports (**Figure 2C**) (32, 90). In contrast to AAV-PHP.eB, AAV.CAP-B10 preferentially targets neurons over astrocytes and oligodendrocytes. No marked discrepancies in viral tropism characterization were observed with single *versus* dual injections.

To quantify the similarity of the AAV tropism characterizations obtained with IHC and scRNA-seq, we directly compared the transduction rate of each AAV variant for every cell type and its corresponding marker gene (i.e., Rbfox3, S100b, or Olig2) as determined by each technique and noticed a good correlation (**Figure 2D**). Despite the different underlying biological readouts–protein expression in IHC and RNA molecules in labeled cell types for scRNA-seq–the two techniques reveal similar viral tropisms.

### 2.3 Tropism Profiling at Transcriptomic Resolution Reveals AAV Variant Biases for Neuronal Subtypes

**Figure 3:**
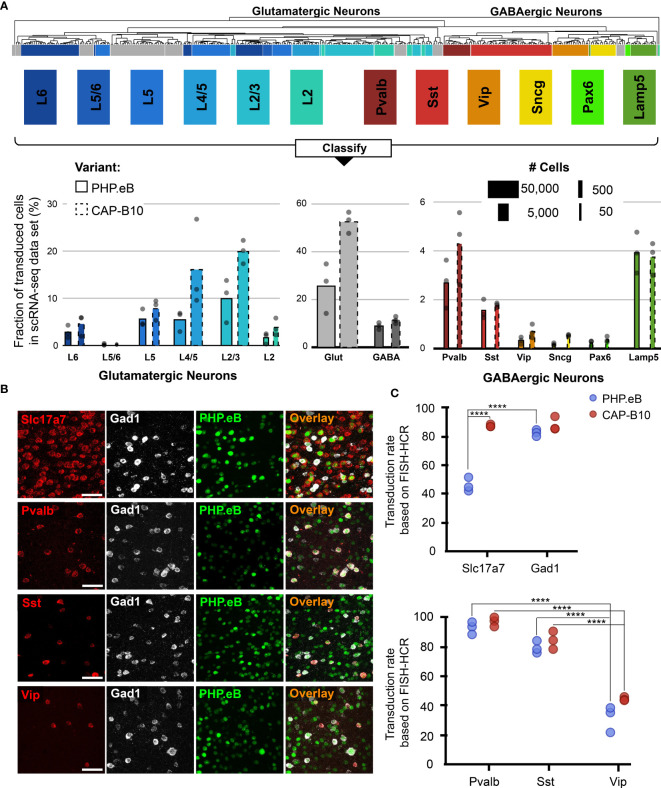
In-depth AAV tropism characterization of neuronal subtypes at transcriptomic resolution. **(A)** Viral tropism profiling across neuronal subtypes. Neuronal subtype annotations are predicted by a model learned from the Allen Institute reference dataset using scANVI (94, 95). Each dot represents data from one animal injected with AAV-PHP.eB and/or AAV.CAP-B10. Bar width indicates the total number of cells of a particular cell type present in our dataset. **(B)** Representative confocal images of cortical tissue from an animal injected with 1.5 × 10^11^ vg of AAV-PHP.eB. Tissue was labeled with FISH-HCR for gene markers of glutamatergic neurons (Slc17a7) and GABAergic neurons (Gad1, Pvalb, Sst, Vip). AAV-PHP.eB shows the endogenous fluorescence of mNeonGreen. Scale bar, 50 µm. **(C)** Confirmation of viral tropism biases across neuronal subtypes using FISH-HCR (3 mice per AAV variant, 1.5 × 10^11^ vg dose). Dots represent the average values across three brain slices from one animal. Results from a two-way ANOVA with correction for multiple comparisons using Tukey’s test is reported with adjusted P-values (****P ≤ 0.0001; and P > 0.05 is not shown on the plot).

After validating our approach against the current standard of AAV tropism characterization (IHC imaging), we scrutinized the tropism of AAV-PHP.eB and AAV.CAP-B10 beyond the major cell types (**Figure 3**). Since AAV.CAP-B10 has increased neuronal bias relative to AAV-PHP.eB, we first sought to understand if there were neuronal subtypes that were differentially responsible for this bias. However, in-depth cell typing of transcriptomes collected from tissues with numerous and complex cell types, such as neurons in the brain, requires expert knowledge of the tissue composition, time to manually curate the data, and the availability of large datasets (66). To minimize the burden of manual annotation, computational tools have been developed that use previously-annotated single-cell databases to predict the cell type of cells in new, unannotated single-cell experiments, even across single-cell platforms (94, 96, 97). We decided to leverage these tools and expanded our marker gene-based cell typing approach by having more complicated or well-established cell types be assigned based on annotations in a reference dataset (**Supplemental Figure 4A**). To this end, we again employed scANVI to construct a joint model of cells from our samples and cells from an annotated reference database. For this model, we used the Mouse Whole Cortex and Hippocampus 10x v2 dataset available from the Allen Brain Institute (95). Since this is a neuron-enriched dataset, we constructed the model using only the 125,341 cells in our dataset classified as neurons from our marker-based QC pipeline combined with the 561,543 neuronal cells from cortical regions from the reference database. We trained this model to predict to which of 14 neuron subtype groupings each cell belonged. We held out 10% of the data for testing: the model performed with 97.9% classification accuracy on the held-out data. We then applied the model to predict the neuron subtypes of our cells.

During our in-depth characterization, we discovered several previously unnoticed cell-subtype biases for AAV-PHP.eB and AAV.CAP-B10 (**Figure 3A**). Starting at the top of our neuronal hierarchy, the fraction of transduced cells that were glutamatergic neurons was markedly reduced for AAV-PHP.eB compared with AAV.CAP-B10 (P = 0.03, 2-sided student t-test, corrected for 2 neuron subtype comparisons). Furthermore, Pvalb+ and Sst+ inhibitory neurons both represented a larger fraction of transduced cells than Vip+ inhibitory neurons (adjusted P = 0.0009, P = 0.045, respectively, two-way ANOVA with multiple comparison correction for inhibitory neuron subtypes using Tukey’s method).

To confirm these tropism biases in neuronal subtypes with a traditional technique, we performed FISH-HCR for glutamatergic and GABAergic gene markers (**Figure 3B**) (98, 99). As indicated by our scRNA-seq data, AAV.CAP-B10, when compared with AAV-PHP.eB, has increased transduction efficiency of glutamatergic neurons (SLC17A7). Furthermore, FISH-HCR verified the downward trend in transduction efficiency from Pvalb+, to Sst+, to Vip+ neurons in both AAV variants (**Figure 3C**).

### 2.4 Pooled AAVs Packaging Barcoded Cargo Recapitulate the Non-Neuronal Tropism Bias of PHP.V1

**Figure 4:**
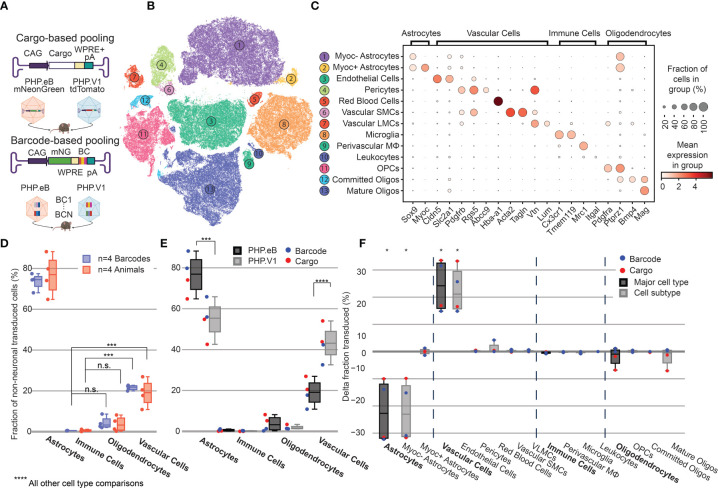
Barcoded co-injected rAAVs reveal the non-neuronal tropism bias of AAV-PHP.V1. **(A)** Experimental design for comparing barcode *vs* cargo-based tropism profiling. Animals received dual injections of AAV-PHP.eB and AAV-PHP.V1, carrying either distinct fluorophores (cargo) or the same fluorophore with distinct barcodes. **(B)** t-SNE projection of the single-cell Variational Inference (scVI) latent space of cells and their cell type classification of the 176,724 labeled non-neuronal cells across all our samples. Each number corresponds to the cell type labeled in **(C)**. **(C)** Marker genes used to identify non-neuronal cell types. Darker colors indicate higher mean expression, and dot size correlates with the abundance of the gene in that cell type. **(D)** The distribution of non-neuronal cells expressing transcripts from AAV-PHP.eB across 4 barcodes within one animal (blue) and across 5 animals (red). All animals received dual injections, with one of the vectors being 1.5 x 10^11^ vg of PHP.eB carrying CAG-mNeonGreen. The y-axis represents the fraction of transduced non-neuronal cells that are of the specified cell type. (***P≤ 0.001 and P > 0.05 (n.s.) is shown; all other cell-type comparisons within a paradigm were significant at P ≤ 0.0001). **(E)** The distribution of non-neuronal cells expressing transcripts from AAV-PHP.eB (black) and AAV-PHP.V1 (gray). Results from the different experimental paradigms are combined. Results shown are from a two-way ANOVA with correction for multiple comparisons using Sidak’s test comparing transduction by AAV-PHP.eB to AAV-PHP.V1 for each cell type, with adjusted P-values (****P ≤ 0.0001, ***P ≤ 0.001 is shown; P > 0.05 is not shown). **(F)** Within-animal difference in the fraction of cells transduced with AAV-PHP.V1 relative to AAV-PHP.eB across four animals, two from each experimental paradigm. For each cell type in each sample, the combined 2-proportion z score for the proportion of that cell type transduced by AAV-PHP.V1 *vs* AAV-PHP.eB is reported. Cell types with fewer than 2 cells transduced by both variants were discarded. Z scores were combined across multiple animals using Stouffer’s method and corrected for multiple comparisons. Cell-type differences with an adjusted P-value below 0.05 are indicated with *.

To enable profiling viral variants in parallel without needing distinct transgenes per variant, we established a barcoding strategy whereby we package AAV variants with the same transgene and regulatory elements but with short, distinguishing nucleotide sequences within the 3’ UTR (**Figure 4A**). To verify that this barcoding strategy can recover tropisms consistent with our previous transgene-based capsid-identification strategy, we performed a set of experiments to re-characterize the tropism of AAV-PHP.eB in parallel with that of the recently developed AAV-PHP.V1, which has increased specificity for vascular cells over AAV-PHP.eB (45).

We produced AAV-PHP.eB carrying CAG-mNeonGreen and AAV-PHP.V1 carrying either CAG-mRuby2 or CAG-tdTomato. Additionally, we produced AAV-PHP.eB and AAV-PHP.V1 both carrying CAG-mNeonGreen with 7-nucleotide barcodes 89 bp upstream of the polyadenylation start site such that they did not interfere with the WPRE. We ensured each barcode had equal G/C content, and that all barcodes were Hamming distance 3 from each other (**Supplemental Table 5**). Each of the barcoded variants was packaged with multiple barcodes that were pooled together during virus production. Four animals received a retro-orbital co-injection of 1.5 x 10^11^ vg/each of AAV-PHP.V1 and AAV-PHP.eB. Two animals received viruses carrying separate fluorophores (cargo-based), and two animals received viruses carrying the barcoded cargo (barcode-based). For amplification of the viral cDNA in the animals receiving the barcoded cargo, we used primers closer to the polyA region such that the sequencing read covered the barcoded region (**Supplemental Table 1**). During the single-cell sequencing dissociation and recovery, one of our dissociations resulted in low recovery of neurons (**Supplemental Figure 4C**); thus, we investigated only non-neuronal cells for this experiment.

Despite variability in the total transgene RNA content between barcodes of the same variant (**Supplemental Figure 5A**), the estimated percent of cells expressing the transgene within each cell type was consistent between barcodes within a single animal, with standard deviations ranging from 0.002 to 0.056 (**Supplemental Figure 6A**). Our analysis of both the barcode-based animals and cargo-based animals shows the same bias in non-neuronal tropism, with AAV-PHP.eB significantly preferring astrocytes over oligodendrocytes, vascular cells, and immune cells (**Figure 4D**). Interestingly, our analysis also revealed that the variance between barcodes within an animal was less than the variance between animals, even when controlling for cargo and dosage (P = 0.030, Bartlett’s test, P-values combined across all variants and cell types using Stouffer’s method, weighted by transduced cell type distribution). This is not surprising, since we found differences in cell type distribution alone can account for up to 58% of the perceived variability in tropism bias (**Supplemental Figure 6B**).

Next, we investigated the distribution of cells transduced by AAV-PHP.eB *vs* AAV-PHP.V1 in the major non-neuronal cell types across both barcode-based and cargo-based paradigms (**Figure 4E**). The single-cell tropism data confirms the previously-established finding that AAV-PHP.V1 has a bias toward vascular cells relative to AAV-PHP.eB. Additionally, we uncovered that this is coupled with a bias away from astrocytes relative to AAV-PHP.eB, but that transduction bias of oligodendrocytes and immune cells did not differ between the variants. To investigate for a specific effect of the barcoding strategy, we performed a three-way ANOVA across the variant, cell type, and experimental paradigm factors. We found that the cell type factor accounted for 87.80% of the total variation, the combined cell type + variant factor accounted for 8.39% of the total variation, and the combined cell type + experimental paradigm factor accounted for only 2.36% of the total variation, confirming our hypothesis that barcoded pools can recover tropism with minimal effect.

### 2.5 Relative Tropism Biases Reveal Non-Neuronal Subtypes With Reduced AAV Transduction

To further characterize the tropism biases of AAV-PHP.V1 and expand our method to less well-established cell hierarchies, we explored the non-neuronal cell types in our dataset. Since the Allen Brain Institute reference database that we used to investigate neuronal tropism was enriched for neurons, it does not contain enough non-neuronal cells to form a robust non-neuronal cell atlas. Our combined dataset consists of 203,661 non-neuronal cells, making it large enough to establish our own non-neuronal cell clustering. Thus, we performed an additional round of automatic clustering on the cells classified as non-neuronal in our combined dataset, and identified 13 non-neuronal cell subtypes based on previously established marker genes (**Figures 4B, C** and **Supplemental Table 2**).

Most cell subtypes had multiple clusters assigned to them, which suggested there may be additional subtypes of cells for which we did not find established marker genes. To determine whether any of these clusters delineated cell types with distinct transcriptional profiles, we investigated the probability of gene expression in each cluster compared to the other clusters of the same cell subtype (see *Methods*). Our approach determined two subclusters of pericytes and astrocytes. Both clusters of pericytes had strong expression of canonical pericytes marker genes Rgs5, Abcc9, and Higd1b. However, one of the clusters had no marker genes that made it distinct from the other pericyte cluster, nor from endothelial cells. Consistent with previous reports, this suggests that this cluster could be pericytes contaminated with endothelial cell fragments, and thus was not considered for further analysis (100–102). Two distinct groups of astrocytes were detected, one of which had unique expression of Myoc and Fxyd6. Using these new marker genes, we expanded our non-neuronal cell taxonomy to 13 cell types, now including Myoc+ and Myoc- astrocytes.

Given our finding that inter-sample variability exceeds intra-sample variability, we established a normalization method for comparing transduction biases between variants co-injected into the same animal. This normalization–calculating the difference in the fraction of transduced cells between variants–captures the relative bias between variants, instead of the absolute tropism of a single variant (see *Methods*). By considering the relative bias between variants, we are able to interrogate tropism in a way that is more robust to inter-sample variability that arises from different distributions of recovered cells, expression rate of delivered cargo, and success of the injection. Using this normalization method, we evaluated the non-neuronal cell type bias of AAV-PHP.V1 relative to AAV-PHP.eB in both the cargo-based animals and the barcode-based animals across our non-neuronal cell-type taxonomy (**Figure 4F**). We discovered that the bias of AAV-PHP.V1 for vascular cells is driven by an increase in transduction of endothelial cells, but not pericytes. Similarly, AAV-PHP.V1’s bias away from astrocytes is driven by a decrease in transduction of Myoc- astrocytes, but not Myoc+ astrocytes. Further inspection of the transduction of pericytes and Myoc+ astrocytes revealed that pericytes are not highly transduced by any of the AAVs tested in this work, and that Myoc+ astrocytes have both lower viral transcript expression and lower abundance than Myoc- astrocytes, and thus do not contribute significantly to tropism (**Supplemental Figures 4, 7A, B**).

### 2.6 Single-Cell RNA Sequencing Reveals Early Cell-Type-Specific Responses to IV Administration of AAV-PHP.eB That Return to Baseline by 3.5 Weeks

**Figure 5:**
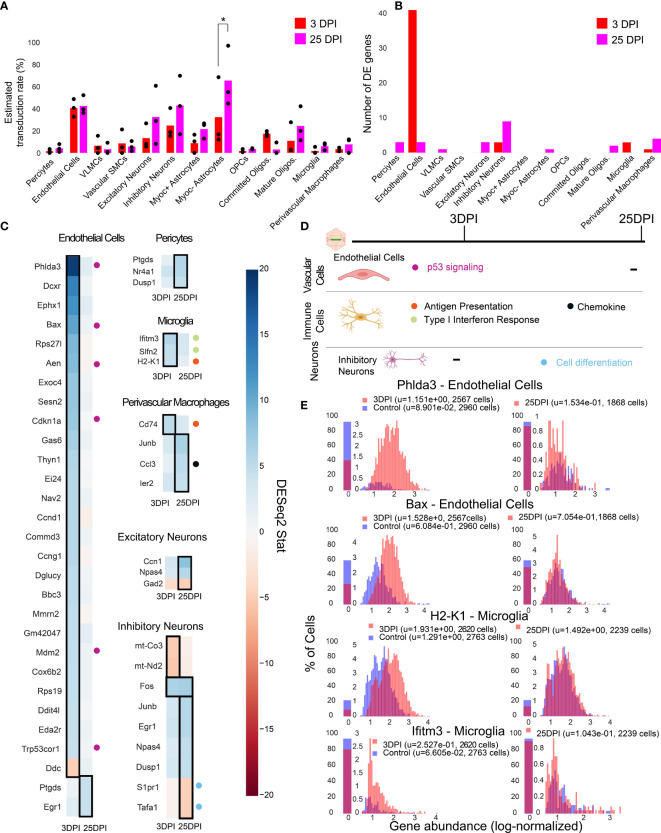
Single-cell gene expression profiling finds cell-type-specific responses to AAV transduction in endothelial cells. **(A)** Estimated transduction rate (%) of mNeonGreen cargo at three and twenty-five days post-injection (DPI). Results from a two-way ANOVA with correction for multiple comparisons using Sidak’s method is reported with adjusted P-values (*P ≤ 0.05 is shown; and P > 0.05 is not shown on the plot). **(B)** Number of differentially expressed genes (adjusted P-value < 0.05, DESeq2) at 3 DPI and 25 DPI across 3 animals. **(C)** Differentially expressed genes across the two time points in endothelial cells, pericytes, microglia, perivascular macrophages, inhibitory neurons, and excitatory neurons. Color indicates DESeq2 test statistic with red representing downregulation and blue representing upregulation. Genes outlined by a black rectangle are determined to have statistically significant differential expression compared to controls (adjusted P-value < 0.05, DESeq2, after Benjamini/Hochberg multiple comparison correction across all cell types and conditions). Colored circles adjacent to each gene indicate the corresponding pathway presented in **(D)**. **(D)** A summary of corresponding pathways in which the differentially regulated genes in **(C)** are involved across the time points. **(E)** Distribution of p53 signaling transcripts in endothelial cells (3 animals are combined) and an example of an MHC-I gene upregulated in microglia at 3 DPI.

To investigate the temporal cell-type-specific transcriptional effects of systemic AAV delivery and cargo expression, we performed a single-cell profiling experiment comparing animals injected with AAV to saline controls. We injected six male mice with AAV-PHP.eB (1.5 x 10^11^ vg) carrying mNeonGreen, and performed single-cell sequencing on three mice three days post-injection (3 DPI) and three mice twenty-five days post-injection (25 DPI). These time points were chosen based on previous work showing MHC presentation response peaking around day seven and transgene response peaking around day 30 (89). Three saline control mice were processed 3 DPI. We then analyzed differential gene expression for each cell type between injected animals and controls using DESeq2 (**Supplemental Table 8**). Of note, we excluded cell types with less than 50 cells in each sample, and excluded leukocytes and red blood cells given the risk of their presence due to dissociation rather than chemokine mediated infiltration. Additionally, we collapsed subtypes of excitatory neurons, inhibitory neurons, and OPCs to have greater than 50 cells for differential expression analysis. We estimated viral transduction rate of AAV-PHP.eB using its delivered cargo, mNeonGreen, across cell types and time points. We identified that Myoc- astrocytes have significantly higher estimated transduction rate at 25 DPI compared to 3 DPI (adjusted P-value = 0.0438, two-way ANOVA with multiple comparison correction using Sidak’s method). It is also worth noting that endothelial cells have a similar transduction rate between the time points in all animals, while one of the animals at 25 DPI exhibited higher transduction in neurons (**Figure 5A**). The number of statistically relevant genes between the injected and control group (adjusted P-value < 0.05, DESeq2) were highest in endothelial cells (41 genes) at 3 DPI, followed by inhibitory neurons (9 genes) at 25 DPI (**Figure 5B**) (adjusted P-value < 0.05, DESeq2).

We found that endothelial cells had the most acute response at 3 DPI with p53 signaling pathway notably impacted. A significant upregulation of Phlda3 and its effectors Bax, Aen, Mdm2, and Cdkn1a, all involved in the p53/Akt signaling pathway, was present (**Figures 5C, E**) (103, 104). Of relevance, we also detected Trp53cor1/LincRNA-p21, responsible for negative regulation of gene expression (105), upregulated in endothelial cells at 3 DPI. Another example of an upregulated gene relevant to inflammation and stress response in endothelial cells is Mmrn2, responsible for regulating angiogenesis in endothelial cells (106).

In brain immune cells, we observe a few substantial changes in genes pertaining to immune regulation at 3 DPI for microglia and at 25 DPI for perivascular macrophages. For example, we observe an upregulation of MHC-I gene H2-K1 at 3 DPI in microglia, which then stabilizes back to control levels at 25 DPI (**Figure 5C**). Ifitm3 and Slfn2, genes implicated in type I interferon response (107, 108), also show upregulation at 3 DPI in microglia. Cd74, a chaperon responsible for regulating antigen presentation during immune response, was upregulated in perivascular macrophages at 3 DPI (109). We did not observe significant differences in pro-inflammatory chemokines, Ccl2 and Ccl5, which are related to breakdown of the blood-brain barrier *via* regulation of tight-junction proteins and recruitment of peripheral leukocytes (110). Ccl3, responsible for infiltration of leukocytes and CNS inflammation (111), was upregulated in perivascular macrophages in 25 DPI (**Figure 5C**).

We found that neurons had only a few differentially expressed genes at 25 DPI. Immediate early genes, such as Fos and Junb were upregulated in inhibitory neurons, while genes involved in modulating cell proliferation, such as Tafa1 and S1pr1, were downregulated at 25 DPI (79, 112).

By investigating the gene expression differences in subpopulations of cells post-injection, we found that endothelial cells upregulate genes linked to p53 signaling at 3 DPI (**Figure 5D**) which all return to control levels at 25 DPI. Immune cells such as microglia and perivascular macrophages upregulate genes involved in type I interferon response, MHC-I antigen processing, and chemokine signaling (**Figure 5D**). Inhibitory neurons display a subtle effect, consisting of differential expression of genes involved in stress response and cell proliferation at 25 DPI.

### 2.7 Larger Pools of Barcoded AAVs Recapitulate Complex Tropism Within a Single Animal

To showcase the capabilities of parallel characterization, we next designed a 7-variant barcoded pool that included the three previously characterized variants (AAV-PHP.eB, AAV-CAP-B10, and AAV-PHP.V1), AAV9 and AAV-PHP.B controls, and two additional variants, AAV-PHP.C1 and AAV-PHP.C2. For simplification of cloning and virus production, we designed a plasmid, UBC-mCherry-AAV-cap-in-cis, that contained both the barcoded cargo, UBC-mCherry, and the AAV9 capsid DNA (**Supplemental Figure 1B**). We assigned three distinct 24 bp barcodes to each variant (**Supplemental Table 5**). Each virus was produced separately to control the dosage, and 1.5 x 10^11^ vg of each variant was pooled and injected into a single animal.

After 3 weeks of expression, we performed single-cell sequencing on extracted cortical tissue. To increase the number of cells available for profiling, we processed two aliquots of cells, for a total of 36,413 recovered cells. To amplify the viral transcripts, we used primers that bind near the 3’ end of mCherry such that the barcode was captured in sequencing (**Supplemental Table 1**).

**Figure 6:**
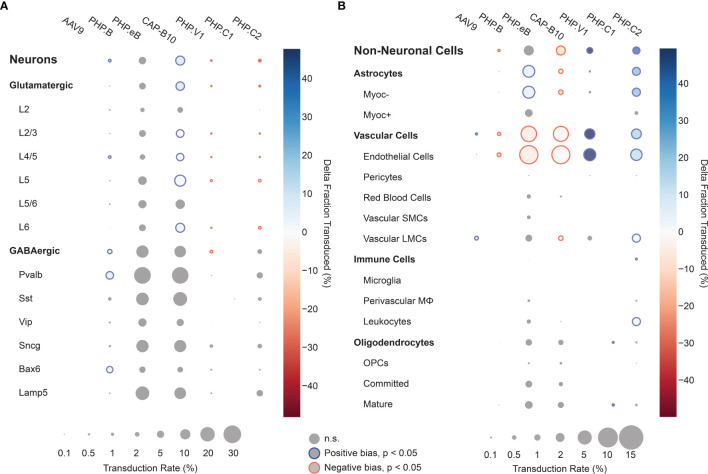
Single animal injections of multiple barcoded rAAVs enables deep, parallel characterization. **(A, B)** Relative cell type tropism of 7 co-injected rAAVs for neuronal **(A)** and non-neuronal **(B)** cell types. The color scale indicates the difference in transduction bias of a variant relative to all other variants in the pool. The area of each circle scales linearly with the fraction of cells of that type with viral transcripts above background. For each variant and cell type, a 2-proportion z score was calculated to compare the number of cells of that type transduced by that variant relative to all other variants combined. Z scores were combined across two single-cell sequencing aliquots using Stouffer’s method and corrected for multiple comparisons. Cell types with fewer than 10 transduced cells in either the variant or variants compared against were discarded. Only cell-type biases at an adjusted P-value < 0.05 are colored; otherwise they are grayed out.

Using our cell typing and viral transcript counting methods, we investigated the transcript counts and transduction bias of the variants in the pool. Compared with our previous profiling experiments, the log-transformed transcript abundance of UBC-mCherry detected per cell was lower than CAG-mNeonGreen-WPRE and CAG-tdTomato (adjusted P < 0.0001, P=0.0767, respectively, two-way ANOVA with multiple comparison correction using Tukey’s method) and shifted towards vascular cells (adjusted P < 0.0001, P=0.0004, respectively, two-way ANOVA with multiple comparison correction using Tukey’s method) (**Supplemental Figures 5B, C**). Next, we looked at the transduction rate difference for each variant compared with the rest of the variants in the pool for each cell type in our taxonomy (**Figures 6A, B**). Despite the lower expression rate and bias shift, the transduction rate difference metric captured the same tropism biases for AAV.CAP-B10 and AAV-PHP.V1 as determined from our previous experiments. AAV.CAP-B10 showed enhanced neuronal targeting relative to other variants in the pool, with this bias coming specifically from an increase in the transduction of glutamatergic neurons. All five variants with transcripts detected in neurons showed a decreased transduction rate in Vip+ neurons relative to other GABAergic neuronal subtypes (**Supplemental Figure 7C**). AAV-PHP.eB showed enhanced targeting of astrocytes (+5.9%, P = 3.0 x 10^-10^, 2-proportion z-test, multiple comparison corrected with Benjamini/Hochberg correction), and AAV-PHP.V1 showed strong bias for vascular cells (+49.7%, p = 1.7 x 10^-45^). In addition to confirming all our existing hypotheses, we were able to identify biases for the previously reported AAV-PHP.C2, which has not been characterized in depth. This variant, which was reported as having a non-neuronal bias similar to AAV-PHP.V1, showed significant transduction bias not only toward vascular cells (+15.7%, P = 1.5 x 10^-7^), but also toward astrocytes (+21.5%, P = 3.0^-28^), and a bias away from neurons (−38%, p = 4.5 x 10^-32^).

## 3 Discussion

The advent of NGS has enabled screening of large libraries of AAV capsids *in vivo* by extracting viral DNA from relevant tissue followed by sequencing of capsid gene inserts or DNA barcodes corresponding to defined capsids. To date, NGS-based screening has been successfully applied to libraries created by peptide insertions (28, 113), DNA shuffling of capsids (114–116), and site-directed mutagenesis (117). Although these NGS-based strategies allow the evolution of new AAV variants with diverse tissue tropisms, it has been difficult to obtain a comprehensive profiling for multiple variants across cell types, which is of utmost importance in organs with complex cell-type compositions, such as the brain (34, 45, 64–66). Towards this end, techniques such as IHC, fluorescent *in situ* RNA hybridization (98, 118–122) or *in situ* RNA sequencing (123–125) can be employed. Several limitations make it challenging to apply these techniques as high-throughput, post-selection AAV tropism profiling methods. First, the limits of optical resolution and the density of transcripts in single cells pose challenges for full *in situ* transcriptome analysis and, until recently, have restricted the total number of simultaneously measured genes in single cells within tissue to several hundred (121, 123–126). By contrast, scRNA-seq with the 10x Genomics Chromium system enables detection of over 4000 genes per cell (95), fast transcriptomic analysis, and multiplexing across different tissue types (127, 128). Furthermore, the method is already widely used by the research community which can help with adoption of our proposed pipelines. Although droplet-based scRNA-seq methods lose spatial information during the dissociation procedure, analysis packages have been developed that can infer single-cell localization by combining scRNA-seq data with pre-existing information from ISH-based labeling for specific marker genes (129–134). Therefore, scRNA-seq techniques have great potential to rapidly profile the tropism of multiple AAV variants in parallel across several thousand cells defined by their entire transcriptome.

Here, we established an experimental and data-analysis pipeline that leverages the capabilities of scRNA-seq to achieve simultaneous characterization of several AAV variants across multiplexed tissue cell types within a single animal. To differentiate multiple AAV capsid variants in the sequencing data, we packaged variants with unique transgenes or the same transgene with unique barcodes incorporated at the 3’ end. We added standard Illumina sequencing primer recognition sites (Read 2) to the viral transcripts using PCR amplification such that the barcoded region could be consistently read out from the Illumina sequencing data. Our computational pipeline demultiplexes viral reads found in the transcriptome according to which matching sequence is most abundant in a separate amplified viral transgene library. Comparing the distribution of viral transcripts by cell type to a null model of empty droplets, we could then determine the cell-type biases.

Our platform has corroborated the tropism of several previously characterized AAV variants and has provided more detailed tropism information beyond the major cell types. The fraction of transduced cells that are glutamatergic neurons was found to be markedly reduced for AAV-PHP.eB when compared with AAV.CAP-B10. Furthermore, within all the variants we tested, both Pvalb+ and Sst+ inhibitory neurons have greater transduction rates than Vip+ neurons. This bodes well for delivery to Pvalb+ neurons, which have been implicated in a wide range of neuro-psychiatric disorders (135), and suggests Vip+ interneurons, which have recently been identified as being a sufficient delivery target for induction of Rett syndrome-like symptoms, as a target for optimization (136). Awareness of neuronal subtype biases in delivery vectors is critical both for neuroscience researchers and for clinical applications. Dissection of neural circuit function requires understanding the roles of neuronal subtypes in behavior and disease and relies on successful and sometimes specific delivery of transgenes to the neuronal types under study (1).

We further discovered that the vascular bias of AAV-PHP.V1 originates from its transduction bias towards endothelial cells. Interestingly, this is the only cell type we detected expressing Ly6a (**Supplemental Figure 8**), a known surface receptor for AAV variants in the PHP.B family (137–139). Given AAV-PHP.V1’s sequence similarity to AAV-PHP.B and its tropism across mouse strains, this pattern suggests that AAV-PHP.V1 transduction may also be Ly6a-mediated. Finding such associations between viral tropism and cell-surface membrane proteins also suggests that full transcriptome sequencing data may hold a treasure trove of information on possible mechanisms of transduction of viral vectors.

We also revealed that AAV-PHP.C2 has a strong, broad non-neuronal bias toward both vascular cells and astrocytes. AAV-PHP.C2 also transduces BALB/cJ mice, which do not contain the Ly6a variant that mediates transduction by PHP.B family variants (137–139). This suggests that PHP.C2 may be the most promising candidate from this pool for researchers interested in delivery to non-neuronal cells with minimal neuronal transduction both in C57BL/6J mice and in strains and organisms that do not have the Ly6a variant.

All our tested variants with non-neuronal transduction have lower expression in Myoc+ astrocytes and pericytes. Astrocytes expressing Myoc and Gfap, which intersect in our data (**Supplemental Figure 8**), have been previously identified as having reactive behavior in disease contexts, making them a target of interest for research on neurological diseases (140, 141). Similarly, pericytes, whose dysfunction has been shown to contribute to multiple neurological diseases, may be an important therapeutic target (60, 142, 143). Both of these cell types may be good candidates for further AAV optimization but may have been missed with marker gene-based approaches. In both AAV characterization and neuroscience research efforts, different marker genes are often used for astrocyte classification – sometimes more restrictive genes such as Gfap, and other times more broadly expressing genes such as S100b or Aldh1l1 (144, 145). Similarly, defining marker genes for pericytes is still an active field (100, 102). Given the constraints of having to choose specific marker genes, it is difficult for staining-based characterizations to provide tropism profiles that are relevant for diverse and changing research needs. This highlights the importance of using unbiased, full transcriptome profiling for vector characterization.

We have shown that our combined experimental and computational platform is able to recover transduction biases and profile multiple variants in a single animal, even amidst the noise of ambient RNA. We have further shown that our method is robust to the variability inherent in delivery and extraction from different animals, with different transgenes, and with different regulatory elements. For example, we discovered lower overall expression from vectors carrying UBC-mCherry compared with CAG-mNeonGreen-WPRE. Such differences are not surprising since the WPRE is known to increase RNA stability and therefore transcript abundance (146). Furthermore, the shift in cell-type bias may come from the UBC promoter, as even ubiquitous promoters such as CAG and UBC have been shown to have variable levels of expression in different cell types (147). Despite these biases, looking at the differences in transduction between variants delivering the same construct within an individual animal reveals the strongest candidate vectors for on-target and off-target cell types of interest. While we show that our method can profile AAVs carrying standard fluorescent cargo, caution is needed when linking differences in absolute viral tropism to changes in capsid composition alone without considering the contribution of the transgene, regulatory elements, and distribution of cell types in recovered tissue. Therefore, for more robust and relative tropism between variants, we found it beneficial to use small barcodes and co-injections of pools of vectors. Our scRNA-seq-based approach is not restricted to profiling capsid variants but can be expanded in the future to screen promoters (148–150), enhancers (151, 152), or transgenes (86, 153), all of which are essential elements requiring optimization to improve gene therapy.

Finally, we have used scRNA-seq to understand how intra-orbital administration of AAV-PHP.eB affects the host cell transcriptome across distinct time points. Results from our study show genes pertaining to the p53 pathway in endothelial cells are differentially expressed 3 days after injection, an effect which vanishes at a later time point of 25 DPI. Though other cell types such as immune cells and neurons had a few differentially expressed genes pertaining to antigen presentation and cell differentiation, respectively, endothelial cells at 3 DPI are the only cells with a profound response signature. The highest number of differentially expressed genes being in endothelial cells suggests that vascular cells could be the initial responders to viral transduction and expression of the transgene. This is supported by Kodali et al., who have shown that endothelial cells are the first to elicit a response to peripheral inflammatory stimulation by transcribing genes for proinflammatory mediators and cytokines (154). With regards to p53 differentially expressed genes, Ghouzzi, et al. have also shown that the genes Phdla3, Aen, and Cdkn1a were upregulated in cells infected with ZIKA virus, signifying genotoxic stress and apoptosis induction (104). Upregulation of genes such as Bax and Cdkn1a could be a response to cellular stress induced by viral transduction (103, 155). However, the initial inflammatory responses did not escalate as shown by the low number of differential expressed genes across all cells (**Figure 5B**) at day twenty-five. Additionally, antigen presenting genes, such as Cd74 and H2-K1, returning back to control expression levels in microglia and a lack of proinflammatory cytokines being upregulated support that the event of infiltration of peripheral leukocytes is unlikely, in agreement with prior studies (87, 88). Based on prior studies, the few genes that are differentially expressed at day 25 in excitatory and inhibitory neurons could also be due to transgene expression rather than the virion (89). Upregulation of immediate early genes such as Fos, Junb, and Ier2 in inhibitory neurons could indicate that the cells which are transduced and expressing viral transcripts could be under increased stress and metabolic demands, either directly in response to transgene expression, or in combination with the stresses of dissociation. For example, c-jun and c-fos were found to be upregulated by lung epithelial cells as part of the response to measles virus (156). Given that scRNA-seq is a sensitive technique, it can be prone to high false discovery rates if not properly controlled. To account for this, we used a highly conservative pseudo-bulk differential expression procedure, which has been shown to minimize false discovery rates compared to other batch correction methods (157). This conservative procedure, however, has lower relative power, and thus there may be additional effects in cell subtypes to AAV transduction. The confidence and statistical power of future scRNA-seq studies looking at AAV-related immune responses could be improved with increased sample size, such as *via* sample multiplexing strategies to pool multiple animals (127), or by increasing the sensitivity and specificity of viral transcript detection and performing differential expression within animals. It is also important to note that the findings discussed here are specific to the rAAV, transgene, and dosage. Nonetheless, our results highlight the power of single-cell profiling in being able to ascertain cell-type-specific responses at an early time point post-injection.

As shown throughout this work, there are several challenges we had to overcome to gain valuable insights from our droplet-based single-cell RNA sequencing approach. While we were able to overcome these in the context of our study, they hint at some important limitations of this method. First, droplet-based single-cell sequencing of tissues that are difficult to dissociate, such as brain, can lead to substantial background noise from debris. Alternative methods, such as single-nucleus RNA sequencing (158, 159), could potentially overcome this debris problem. Exploratory work would need to be performed to determine whether single-nucleus RNA sequencing captures a sufficient amount of immature viral transcripts, but, if effective, may obviate the need for computational detection of transduction above a background level. Another potential challenge of our method is scaling up to much larger numbers of variants. In order to establish high statistical confidence in tropism, many cells need to be transduced. However, given restrictions on the total dosage an animal can receive, adding more variants would require a lower dosage per variant. In simulation, we found that subsampling our 2-variant pool by 10-fold did not change the major tropism findings (**Supplemental Figure 9**). Given our current injections are 8-fold lower than the maximum allowed dosage, this suggests this method could scale up to 80 variants; however, further work would need to be done to validate whether this holds for a diversity of variants that may be competing for binding. Scaling higher would be challenging with current droplet-based single-cell RNA sequencing pipelines that process on the order of 10^4^ cells per reaction. Alternative approaches, such as split-pool strategies, which can profile many more cells (160), may thus be appealing for larger variant pools.

In summary, our platform enables thorough tropism characterization of existing and emerging recombinant AAVs and helps uncover cellular responses to rAAV-mediated gene therapy, thus further guiding the engineering and use of gene delivery vehicles.

## 4 Materials and Methods

### 4.1 Animals

Male C57BL/6J mice (Stock No: 000664) used in this study were purchased from the Jackson Laboratory (JAX). AAV variants were injected i.v. into the retro-orbital sinus of 6–7 week old mice.

### 4.2 Plasmids

*In vivo* vector characterization of AAV variant capsids was conducted using single-stranded (ss) rAAV genomes. pAAV : CAG-NLS-mNeonGreen, pAAV : CAG-NLS-mRuby2, pAAV : CAG-tdTomato, and pAAV : CAG-NLS-tdTomato constructs were adapted from previous publications (32, 45). To introduce barcodes into the polyA region of CAG-NLS-mNeonGreen, we digested the plasmid with BglII and EcoRI, and performed Gibson assembly (E2611, NEB) to insert synthesized fragments with 7bp degenerate nucleotide sequences 89 bp upstream of the polyadenylation site. We then seeded bacterial colonies and selected and performed Sanger sequencing on the resulting plasmids to determine the corresponding barcode.

The UBC-mCherry-AAV-cap-in-cis plasmid was adapted from the rAAV-Cap-in-cis-lox plasmid from a previous publication (34). We performed a restriction digest on the plasmid with BsmbI and SpeI to remove UBC-mCherry and retain the AAV9 cap gene and remaining backbone. We then circularized the digested plasmid using a gblock joint fragment to get a plasmid containing AAV2-Rep, AAV9-Cap, and the remaining backbone *via* T4 ligation. In order to insert UBC-mCherry with the desired orientation and location, we amplified its linear segment from the original rAAV-Cap-in-cis-lox plasmid. The linear UBC-mCherry-polyA segment and circularized AAV2-Rep,AAV9-cap plasmid were then both digested with HindIII and ligated using T4 ligation. In order to get the SV40 PolyA element in the proper orientation with respect to the inserted UBC-mCherry, we removed the original segment from the plasmid using AvrII and AccI enzymes and inserted AvrII, AccI treated SV40 gblock using T4 ligation to get the final plasmid.

To insert barcodes into UBC-mCherry-AAV-cap-in-cis, we obtained 300 bp DNA fragments containing the two desired capsid mutation regions for each variant and the variant barcode, flanked by BsrGI and XbaI cut sites. The three segments of the fragment were separated by BsaI Type I restriction sites. We digested the UBC-mCherry-AAV-cap-in-cis plasmid with BsrGI and XbaI, and ligated each variant insert to this backbone. Then, to reinsert the missing regions, we performed Golden Gate assembly with two inserts and BsaI-HF.

### 4.3 Viral Production

To produce viruses carrying in trans constructs, we followed established protocols for the production of rAAVs (161). In short, HEK293T cells were triple transfected using polyethylenimine (PEI) with three plasmids: pAAV (see *Plasmids*), pUCmini-iCAP-PHP.eB (32), pUCmini-iCAP-CAP-B10 (90), or pUCmini-iCAP-PHP.V1 (45), and pHelper. After 120 h, virus was harvested and purified using an iodixanol gradient (Optiprep, Sigma). For our 7-variant pool, we modified the protocol to be a double transfection using PEI with two plasmids: UBC-mCherry-AAV-cap-in-cis and pHelper.

### 4.4 Tissue Processing for Single-Cell Suspension

Three to four weeks after the injection, mice (9-10 weeks old) were briefly anesthetized with isoflurane (5%) in an isolated plexiglass chamber followed by i.p. injection of euthasol (100 mg/kg). The following dissociation procedure of cortical tissue into a single-cell suspension was adapted with modifications from a previous report (162). Animals were transcardially perfused with ice-cold carbogenated (95% O_2_ and 5% CO_2_) NMDG-HEPES-ACSF (93 mM NMDG, 2.5 mM KCl, 1.2 mM NaH_2_PO_4_, 30 mM NaHCO_3_, 20 mM HEPES, 25 mM glucose, 5 mM Na L-ascorbate, 2 mM thiourea, 3 mM Na-pyruvate, 10 mM MgSO_4_, 1 mM CaCl_2_, 1 mM kynurenic acid Na salt, pH adjusted to 7.35 with 10N HCl, osmolarity range 300–310 mOsm). Brains were rapidly extracted and cut in half along the anterior-posterior axis with a razor blade. Half of the brain was used for IHC histology while the second half of the brain was used for scRNA-seq. Tissue used for scRNA-seq was immersed in ice-cold NMDG-HEPES-ACSF saturated with carbogen. The brain was sectioned into 300-μm slices using a vibratome (VT-1200, Leica Biosystems, IL, USA). Coronal sections from Bregma −0.94 mm to −2.80 mm were collected in a dissection dish on ice containing NMDG-HEPES-ACSF. Cortical tissue from the dorsal surface of the brain to ~3.5 mm ventral was cut out and further sliced into small tissue pieces. NMDG-HEPES-ACSF was replaced by trehalose-HEPES-ACSF (92 mM NaCl, 2.5 mM KCl, 1.2 mM NaH_2_PO_4_, 30 mM NaHCO_3_, 20 mM HEPES, 25 mM glucose, 2 mM MgSO_4_, 2 mM CaCl_2_, 1 mM kynurenic acid Na salt, 0.025 mM D-(+)-trehalose dihydrate*2H_2_O, pH adjusted to 7.35, osmolarity ranging 320–330 mOsm) containing papain (60 U/ml; P3125, Sigma Aldrich, pre-activated with 2.5 mM cysteine and a 0.5–1 h incubation at 34°C, supplemented with 0.5 mM EDTA) for the enzymatic digestion. Under gentle carbogenation, cortical tissue was incubated at 34°C for 50 min with soft agitation by pipetting every 10 min. 5 μl 2500 U/ml DNase I (04716728001 Roche, Sigma Aldrich) was added to the single-cell suspension 10 min before the end of the digestion. The solution was replaced with 200 μl trehalose-HEPES-ACSF containing 3 mg/ml ovomucoid inhibitor (OI-BSA, Worthington) and 1 μl DNase I. At room temperature, the digested cortical tissue was gently triturated with fire-polished glass Pasteur pipettes for three consecutive rounds with decreasing pipette diameters of 600, 300, and 150 μm. 800 μl of trehalose-HEPES-ACSF with 3 mg/ml ovomucoid inhibitor was added. The uniform single-cell suspension was pipetted through a 40 μm cell strainer (352340, Falcon) into a new microcentrifuge tube followed by centrifugation at 300 g for 5 min at 4°C. The supernatant was discarded and cell pellet was resuspended in 1 ml of trehalose-HEPES-ACSF. After mixing using a Pasteur pipette with a 150 μm tip diameter, the single-cell suspension was centrifuged again. Supernatant was replaced with fresh trehalose-HEPES-ACSF and the resuspended cell pellet was strained with a 20 μm nylon net filter (NY2004700, Millipore). After resuspension in trehalose-HEPES-ACSF, cells were pelleted again and resuspended in 100 μl of ice-cold resuspension-ACSF (117 mM NaCl, 2.5 mM KCl, 1.2 mM NaH_2_PO_4_, 30 mM NaHCO_3_, 20 mM HEPES, 25 mM glucose, 1 mM MgSO_4_, 2 mM CaCl_2_, 1 mM kynurenic acid Na salt and 0.05% BSA, pH adjusted to 7.35 with Tris base, osmolarity range 320–330 mOsm). Cells were counted with a hemocytometer and the final cell densities were verified to be in the range of 400–2,500 cells/μl. The density of single-cell suspension was adjusted with resuspension-ACSF if necessary.

### 4.5 Transcriptomic Library Construction

Cell suspension volumes containing 16,000 cells–expected to retrieve an estimated 10,000 single-cell transcriptomes–were added to the 10x Genomics RT reaction mix and loaded to the 10x Single Cell Chip A (230027, 10x Genomics) for 10x v2 chemistry or B (2000168, 10x Genomics) for 10x v3 chemistry per the manufacturer’s protocol (Document CG00052, Revision F, Document CG000183, Revision C, respectively). We used the Chromium Single Cell 3’ GEM and Library Kit v2 (120237, 10x genomics) or v3 (1000075, 10x Genomics) to recover and amplify cDNA, applying 11 rounds of amplification. We took 70 ng to prepare Illumina sequencing libraries downstream of reverse transcription following the manufacturer’s protocol, applying 13 rounds of sequencing library amplification.

### 4.6 Viral Library Construction

We selectively amplified viral transcripts from 15 ng of cDNA using a cargo-specific primer binding to the target of interest and a primer binding the partial Illumina Read 1 sequence present on the 10x capture oligos (**Supplemental Table 1**). For animals injected with a single cargo, amplification was performed only once using the primer for the delivered cargo; for animals with distinct cargo sequences per variant, amplification was performed in parallel reactions from the same cDNA library using different cargo-specific primers for each reaction. We performed the amplification using 2x KAPA HiFi HotStart ReadyMix (KK2600) for 28 cycles at an annealing temperature of 53°C. Afterwards, we performed a left-sided SPRI cleanup with a concentration dependent on the target amplicon length, in accordance with the manufacturer’s protocol (SPRISelect, Beckman Coulter B23318). We then performed an overhang PCR on 100 ng of product with 15 cycles using primers that bind the cargo and the partial Illumina Read 1 sequence and appending the P5/P7 sequences and Illumina sample indices. We performed another SPRI cleanup, and analyzed the results *via* an Agilent High Sensitivity DNA Chip (Agilent 5067-4626).

### 4.7 Sequencing

Transcriptome libraries were pooled together in equal molar ratios according to their DNA mass concentration and their mean transcript size as determined *via* bioanalyzer. Sequencing libraries were processed on Novaseq 6000 S4 300-cycle lanes. The run was configured to read 150 bp from each end. Sequencing was outsourced to Fulgent Genetics and the UCSF Center for Advanced Technology.

All viral transcript libraries except barcoded UBC-mCherry were pooled together in equal molar ratios into a 4 nM sequencing library, then diluted and denatured into a 12 pM library as per the manufacturer’s protocol (Illumina Document #15039740v10). The resulting library was sequenced using a MiSeq v3 150-cycle reagent kit (MS-102-3001), configured to read 91 base pairs for Read 2 and 28 base pairs for Read 1. To characterize the effect of sequencing depth, one viral transcript library was additionally processed independently on a separate MiSeq run.

The UBC-mCherry viral transcript library, which was recovered with primers near the polyadenylation site, consisted of fragments ~307 bp long. Since this length is within the common range for an Illumina NovaSeq run, this viral transcript library was pooled and included with the corresponding transcriptome library.

### 4.8 Transcriptome Read Alignment

For transcriptome read alignment and gene expression quantification, we used 10x Cell Ranger v5.0.1 with default options to process the FASTQ files from the transcriptome sequencing library. The reads were aligned against the mus musculus reference provided by Cell Ranger (mm10 v2020-A, based on Ensembl release 98).

To detect viral transcripts in the transcriptome, we ran an additional alignment using 10x Cell Ranger v5.0. 1 with a custom reference genome based on mm10 v2020-A. We followed the protocol for constructing a custom Cell Ranger reference as provided by 10x Genomics. This custom reference adds a single gene containing all the unique sequences from our delivered plasmids in the study, delineated as separate exons. Sequences that are common between different cargo are provided only once, and annotated as alternative splicings.

### 4.9 Viral Transcript Read Alignment

For viral read alignment, we aligned each Read 2 to a template derived from the plasmid, excluding barcodes. The template sequence was determined by starting at the ATG start site of the XFP cargo and ending at the AATAAA polyadenylation stop site. We used a Python implementation of the Striped Smith-Waterman algorithm from scikit-bio to calculate an alignment score for each read, and normalized the score by dividing by the maximum possible alignment score for a sequence of that length, minus the length of the barcode region. For each Read 2 that had a normalized alignment score of greater than 0.7, we extracted the corresponding cell barcode and UMI from Read 1, and any insertions into the template from Read 2.

### 4.10 Constructing the Variant Lookup Table

For co-injections with multiple templates and injections of barcoded templates, we constructed a lookup table to identify which variant belongs to each cell barcode/UMI. For each template, we counted the number of reads for each cell barcode/UMI. For reads of barcoded cargo, we only counted reads where the detected insertion in the barcode region unambiguously aligned to one of the pre-defined variant barcodes. Due to sequencing and PCR amplification errors, most cell barcode/UMI combinations had reads associated with multiple variants. Thus, we identified the variant with the largest count for each cell barcode/UMI. We discarded any cell barcode/UMIs that had more than one variant tied for the largest count. Finally, each cell barcode/UMI that was classified as a viral transcript in the transcriptome (see *Transcriptome Read Alignment*) was converted into the virus detected in the variant lookup table, or was discarded if it did not exist in the variant lookup table.

### 4.11 Estimating Transduction Rate

To determine an estimate of the percent of cells within a group expressing viral cargo above background, we compared the viral transcript counts in that group of cells to a background distribution of viral transcript counts in debris (see *Droplet Type Classification*). First, we obtained the empirical distribution of viral transcript counts by extracting the viral counts for that variant in cell barcodes classified as the target cell type as well as cell barcodes classified as debris. Next, we assumed a percentage of cells containing debris. For each viral transcript count, starting at 0, we calculated the number of cells that would contain this transcript count, if the assumed debris percentage was correct. We then calculated an error between this estimate and the number of cells with this transcript count in the cell type of interest. We tallied this error over all the integer bins in the histogram, allowing the error in a previous bin to roll over to the next bin. We repeated this for all possible values of percentage of debris from 0 to 100 in increments of 0.25, and the value that minimized the error was the estimated percentage of cells whose viral transcript count could be accounted for by debris. The inverse of this was our estimate of the number of cells expressing viral transcripts above background.

To validate that this method reliably recovers an estimate of transduction rate, we performed a series of simulations using models of debris viral transcript counts added to proposed cell type transcript count distributions across a range of parameterizations. To get estimates of the background distribution of debris, we used diffxpy (https://github.com/theislab/diffxpy) to fit the parameters of a negative binomial distribution to the viral transcript counts in debris droplets within a sample. We then postulated 1,000 different parameterizations of the negative binomial representing transcript counts in groups of cells, with 40 values of r ranging from 0.1 to 10, spaced evenly apart, and 25 values of p ranging from 0.001 to 0.99, spaced evenly apart. For each proposed negative binomial model, we drew 1,000 random samples of viral counts from the learned background distribution, and 1,000 random samples from the proposed cell distribution, and summed the two vectors. This summed vector was then used in our transduction rate estimation function, along with a separate 1,000 random samples of background viral transcripts for the function to use as an estimate of the background signal. We calculated the true probability of non-zero expression in our proposed cell negative binomial model (1 – P(X = 0)), and compared this value with the estimated value from the transduction rate estimation method.

### 4.12 Calculating Viral Tropism

For each variant *v_n_* and cell type of interest *c_i_*, we estimated the percentage of cells expressing viral cargo. To calculate tropism bias, we used this estimated expression rate, $t_{c_{i},v_{n}}$ , to estimate the number of cells expressing viral transcripts in that cell type, $T_{c_{i},v_{n}}$ out of the total number of cells of that type, $N_{c_{i}}.\;T_{c_{i},v_{n}}=t_{c_{i},v_{n}}N_{c_{i}}$ . Cell type bias, $b_{c_{i},v_{n}},$ within a sample was then calculated as the ratio of the number of cells of interest divided by the total number of transduced cells, $b_{c_{i},v_{n}}=\frac{T_{c_{i},v_{n}}}{\Sigma_{j}T_{c_{j},v_{n}}}$ . Finally, to calculate the difference in transduction bias for a particular variant relative to other variants in the sample, $\delta_{c_{i},v_{n}}$ , we subtracted the bias of the variant from the mean bias across all other variants, $\delta_{c_{i},v_{n}}=\frac{T_{c_{i},v_{n}}}{\Sigma_{j}T_{c_{j},v_{n}}}-\frac{\Sigma_{m\neq n}T_{c_{i},v_{m}}}{\Sigma_{m\neq n}\Sigma_{j}T_{c_{j},v_{m}}}$ .

### 4.13 Histology

#### 4.13.1 Immunohistochemistry

The immunohistochemistry procedure was adapted from a previous publication (163). Brain tissue was fixed in 4% paraformaldehyde (PFA) at 4°C overnight on a shaker. Samples were immersed in 30% sucrose in 1x phosphate buffered saline (PBS) solution for >2 days and then embedded in Tissue-Tek O.C.T. Compound (102094-104, VWR) before freezing in dry ice for 1 h. Samples were sectioned into 50 μm coronal slices on a cryostat (Leica Biosystems). Brain slices were washed once with 1x phosphate buffered saline (PBS) to remove O.C.T. Compound. Samples were then incubated overnight at 4°C on a shaker in a 1x PBS solution containing 0.1% Triton X-100, 10% normal goat serum (NGS; Jackson ImmunoResearch, PA, USA), and primary antibodies. Sections were washed three times for 15 min each in 1x PBS. Next, brain slices were incubated at 4°C overnight on a shaker in a 1x PBS solution containing 0.1% Triton X-100, 10% NGS, and secondary antibodies. Sections were washed again three times for 15 min each in 1x PBS. Finally, slices were mounted on glass microscope slides (Adhesion Superfrost Plus Glass Slides, #5075-Plus, Brain Research Laboratories, MA, USA). After the brain slices dried, DAPI-containing mounting media (Fluoromount G with DAPI, 00-4959-52, eBioscience, CA, USA) was added before protecting the slices with a cover glass (Cover glass, #4860-1, Brain Research Laboratories, MA, USA). Confocal images were acquired on a Zeiss LSM 880 confocal microscope (Zeiss, Oberkochen, Germany). The following primary antibodies were used: rabbit monoclonal to NeuN (Rbfox3) (1:500; ab177487; Abcam, MA, USA), rabbit monoclonal to S100 beta (1:500; ab52642; Abcam, MA, USA), and rabbit monoclonal to Olig2 (1:500; ab109186; Abcam, MA, USA). The following secondary antibody was used: goat anti-rabbit IgG H&L Alexa Fluor 647 (1:500; ab150079; Abcam, MA, USA).

#### 4.13.2 Fluorescent *In Situ* Hybridization Chain Reaction

FISH-HCR was conducted as previously reported (99). Probes targeting neuronal markers were designed using custom-written software (https://github.com/GradinaruLab/HCRprobe). Probes contained a target sequence of 20 nucleotides, a spacer of 2 nucleotides, and an initiator sequence of 18 nucleotides. Criteria for the target sequences were: (1) a GC content between 45%–60%, (2) no nucleotide repeats more than three times, (3) no more than 20 hits when blasted, and (4) the ΔG had to be above –9 kcal/mol to avoid self-dimers. Last, the full probe sequence was blasted and the Smith-Waterman alignment score was calculated between all possible pairs to prevent the formation of cross-dimers. In total, we designed 26 probes for Gad1, 20 probes for Vip, 22 probes for Pvalb, 18 probes for Sst, and 28 probes for Slc17a7. Probes were synthesized by Integrated DNA Technologies.

### 4.14 Droplet Type Identification

scRNA-seq datasets were analyzed with custom-written scripts in Python 3.7.4 using a custom fork off of scVI v0.8.1, and scanpy v1.6.0. To generate a training dataset for classifying a droplet as debris, multiplets, neuronal, or non-neuronal cells, we randomly sampled cells from all 27 cortical tissue samples. We sampled a total of 200,000 cells, taking cells from each tissue sample proportional to the expected number of cells loaded into the single-cell sequencing reaction. Within each sample, cells were drawn randomly, without replacement, weighted proportionally by their total number of detected UMIs. For each sample, we determined a lower bound on the cutoff between cells and empty droplets by constructing a histogram of UMI counts per cell from the raw, unfiltered gene count matrix. We then found the most prominent trough preceding the first prominent peak, as implemented by the scipy peak_prominences function. We only sampled from cells above this lower bound. Using these sampled cells, we trained a generative neural network model *via* scVI with the following parameters: 20 latent features, 2 layers, and 256 hidden units. These parameters were chosen from a coarse hyperparameter optimization centered around the scVI default values (**Supplemental Table 3**). We included the sample identifier as the batch key so that the model learned a latent representation with batch correction.

After training, Leiden clustering was performed on the learned latent space as implemented by scanpy. We used default parameters except for the resolution, which we increased to 2 to ensure isolation of small clusters of cell multiplets. Using the learned generative model, we draw 5000 cells from the posterior distribution based on random seed cells in each cluster. We draw an equal number conditioned on each batch. From these samples, we then calculated a batch-corrected probability of each cluster expressing a given marker gene (see *Cluster Marker Gene Determination*). For this coarse cell typing, we chose a single marker gene for major cell types expected in the cortex (**Supplemental Table 2**). If a cluster was expressing the neuron marker gene Rbfox3, it was labeled as “Neurons”. If a cluster was expressing any of the other non-neuronal marker genes, it was labelled as “Non-neurons”. Next, we ran Scrublet on the training cells to identify potential multiplets. Scrublet was run on each sample independently, since it is not designed to operate on combined datasets with potential batch-specific confounds. We then calculated the percentage of droplets in each cluster of the combined data that were identified as multiplets by Scrublet. We found a percentage threshold for identifying a cluster as containing predominantly multiplets by using Otsu’s threshold, as implemented by scikit-image. All droplets in any cluster above the multiplet percentage threshold were labelled as “Multiplets”. All other clusters were labelled as “Debris”.

Next, we trained a cell-type classifier using scANVI on the droplets labeled as training data. We used the weights from the previously trained scVI model as the starting weights for scANVI. Rather than using all cells for every epoch of the trainer, we implemented an alternative sampling scheme that presented each cell type to the classifier in equal proportions. Once the model was trained, all cells above the UMI lower noise bound were run through the classifier to obtain their cell-type classification. Droplets classified as “Neurons” or “Non-neurons” were additionally filtered by their scANVI-assigned probability. We retained only cells above an FDR threshold of 0.05, corrected for multiple comparisons using the Benjamini-Hochberg procedure. Finally, since the original run of Scrublet for multiplet detection was performed on only the training data, and thus did not take advantage of all the cells available, we ran Scrublet on all droplets classified as cells, and removed any identified multiplets.

### 4.15 Cluster Marker Gene Determination

To identify which clusters are expressing marker genes, we determined an estimated probability of a marker gene being expressed by a random cell in that cluster. For each cluster, we randomly sampled 5,000 cells, with replacement. We used scVI to project each cell into its learned latent space, and then used scVI’s posterior predictive sampling function to generate an example cell from this latent representation, and tallied how many times the gene is expressed. We repeated this for each batch, conditioning the posterior sample on that batch, to account for technical artifacts such as sequencing depth. Once we obtained a probability of expression of a marker gene for each cluster, we find a threshold for expression using Otsu’s method, as implemented by scikit-image. Clusters that have a probability of expression above the threshold are considered positive for that marker gene.

### 4.16 Neuronal Subtype Classification

Cells classified as neurons were further subtyped using annotations from a well-curated reference dataset. We used the Mouse Whole Cortex and Hippocampus 10x dataset from the Allen Institute for Brain Science as our reference dataset (95). First, we filtered the reference dataset to contain only cell types that are found within the brain regions collected for our experiments. To ensure that, overall, enough cells per cell type were present in our datasets, we merged cell types with common characteristics, such as expression of key marker genes. We re-aligned our cell transcriptome reads to the same pre-mRNA reference used to construct the reference dataset, so that the gene count matrices had a 1:1 mapping. We then trained a joint scANVI model with all cells identified as neurons from our samples and the reference database to learn a common latent space between them. The model was trained to classify cells based on the labels provided in the reference dataset. Cells were sampled from each class in equal proportions during training. After the model was trained, all neurons from our sample were run through the model to obtain their cell type classification.

### 4.17 Non-Neuronal Subtype Classification

Cells classified as non-neuronal were further subtyped using automatic clustering and marker gene identification. We trained an scVI model using only the non-neuronal cells and performed Leiden clustering as implemented by scanpy on the latent space. We determined which clusters were expressing each of 31 marker genes across 13 cell subtypes. Marker genes were identified from a review of existing scRNA-seq, bulk RNA-seq, or IHC studies of mouse brain non-neuronal subtypes (**Supplemental Table 2**). Each cluster was assigned to a cell subtype if it was determined positive for all the marker genes for that cell subtype (see *Cluster Marker Gene Determination*). If a cluster contained all the marker genes for multiple cell subtypes, the cluster was assigned to the cell subtype with the greatest number of marker genes. Clusters that did not express all the marker genes for any cell subtype were labeled as “Unknown”. Clusters that expressed all the marker genes for multiple cell subtypes with the same total number of marker genes were labeled as “Multiplets”. For cell types that contained multiple clusters, we then calculated the probability of every gene being zero in each cluster (see *Cluster Marker Gene Determination*). We then compared gene presence between clusters of the same cell type to see if there were any subclusters that had a dominant marker gene (present in > 50% of samples), that was not present in any of the other clusters (< 10% of samples). For the three cell types that had unique marker genes, we named the cluster after the gene with the highest 2-proportion z-score between the sampled gene counts in that cluster *vs* the rest.

### 4.18 Quantification of Images

Quantitative data analysis of confocal images was performed blind with regard to AAV capsid variant. Manual quantification was performed using the Cell Counter plugin, present in the Fiji distribution of ImageJ (National Institutes of Health, Bethesda, MD) (164). Transduction rate was calculated as the total number of double positive cells (i.e. viral transgene and cell type marker) divided by the total number of cell type marker labeled cells. For each brain slice, at least 100 cells positive for the gene markers of interest were counted in the cortex.

### 4.19 Differential Expression

To calculate differential expression within cell types between groups of animals, we used the DESeq2 R package (165). For each cell type, the gene counts are summed across all cells of that type and treated as a pseudo-bulk sample. The summed gene counts from each animal are then included as individual columns for a DESeq2 differential expression analysis. We performed DE for 3 DPI and 25 DPI separately, testing each sample against saline-injected controls. For each cell type, only genes that were present in all samples of at least one condition are included.

### 4.20 Marker Gene Dot Plots

To generate dot plots for marker genes, we used scanpy’s dotplot function (166). Gene counts were normalized to the sum of the total transcript counts per cell using scanpy’s normalize_total function. Normalized gene expression values are log-transformed as part of the plotting function.

### 4.21 Statistics

Statistical analyses comparing the fraction of transduced cells and transduction rate in different cell types for **Figures 2**, **3**, **4D, E** and **5A** were conducted using GraphPad Prism 9. Statistical analyses comparing proportions of transduced cells within an animal in **Figures 4F** and **Figure 6** were performed using the Python statsmodels library v0.12.1. No statistical methods were used to predetermine sample sizes. The statistical test applied, sample sizes, and statistical significant effects are reported in each figure legend. The significance threshold was defined as a = 0.05.

## Data Availability Statement

All raw FASTQ files are available under the SRA BioProject PRJNA758711 (https://www.ncbi.nlm.nih.gov/bioproject/PRJNA758711/). Processed gene count matrices for droplets identified as cells, as well as the demultiplexed virus cargo counts, are available at CaltechData, doi: 22002/D1.2090 (http://dx.doi.org/10.22002/D1.2090).

## Ethics Statement

Animal husbandry and all experimental procedures involving animals were performed in accordance with the California Institute of Technology Institutional Animal Care and Use Committee (IACUC) guidelines and reviewed and approved by the Office of Laboratory Animal Resources at the California Institute of Technology (animal protocol no. 1650).

## Author Contributions

DB, MA, TD, and VG conceived the project and designed the experiments. SC and MT provided critical single-cell RNA sequencing expertise. TD, MA, and DB prepared the DNA constructs and produced virus. MA performed the injections, tissue dissociation, histology, imaging and image quantification. DB and TD performed the single-cell library preparation and prepared samples for sequencing. DB and MA built the data processing pipeline. DB, MA, TD, and AW performed the analysis. All authors contributed to the MS as drafted by DB, MA, and VG. MT supervised single-cell RNA sequencing computational pipelines while VG supervised the overall project. All authors contributed to the article and approved the submitted version.

## Funding

This work was supported by the NIH Pioneer DP1OD025535, NIH BRAIN R01MH117069, Beckman Institute for CLARITY, Optogenetics and Vector Engineering Research (CLOVER) at Caltech, the Single-Cell Profiling and Engineering Center (SPEC) in the Beckman Institute at Caltech, the Curci Foundation, the CZI Neurodegeneration Challenge Network (VG), and the Vallee Foundation (VG). VG and MT are Heritage Principal Investigators supported by the Heritage Medical Research Institute. DB was supported by PHS Grant Number 5T32NS105595-02.

## Conflict of Interest

VG is a Co-founder and BoD member for Capsida Biotherapeutics, a Fully Integrated AAV Engineering and Gene Therapy Company in Southern California.

The remaining authors declare that the research was conducted in the absence of any commercial or financial relationships that could be construed as a potential conflict of interest.

## Publisher’s Note

All claims expressed in this article are solely those of the authors and do not necessarily represent those of their affiliated organizations, or those of the publisher, the editors and the reviewers. Any product that may be evaluated in this article, or claim that may be made by its manufacturer, is not guaranteed or endorsed by the publisher.

## References

[1] Bedbrook CN Deverman BE Gradinaru V. Viral Strategies for Targeting the Central and Peripheral Nervous Systems. Annu Rev Neurosci (2018) 41(1):323–48. doi: 10.1146/annurev-neuro-080317-062048 29709207

[2] Samulski RJ Muzyczka N. AAV-Mediated Gene Therapy for Research and Therapeutic Purposes. Annu Rev Virol (2014) 1(1):427–51. doi: 10.1146/annurev-virology-031413-085355 26958729

[3] Daya S Berns KI. Gene Therapy Using Adeno-Associated Virus Vectors. Clin Microbiol Rev (2008) 21(4):583–93. doi: 10.1128/CMR.00008-08 PMC2570152 18854481

[4] Deverman BE Ravina BM Bankiewicz KS Paul SM Sah DWY. Gene Therapy for Neurological Disorders: Progress and Prospects. Nat Rev Drug Discovery (2018) 17(9):641–59. doi: 10.1038/nrd.2018.110 30093643

[5] Gaj T Epstein BE Schaffer DV. Genome Engineering Using Adeno-Associated Virus: Basic and Clinical Research Applications. Mol Ther (2016) 24(3):458–64. doi: 10.1038/mt.2015.151 PMC4786909 26373345

[6] Hirsch ML Samulski RJ. AAV-Mediated Gene Editing *via* Double-Strand Break Repair. In: Storici F, editor. Gene Correction: Methods and Protocols. Totowa, NJ: Humana Press (2014). p. 291–307. Available at: https://doi.org/10.1007/978-1-62703-761-7_19. 10.1007/978-1-62703-761-7_19 PMC4971580 24557911

[7] Naso MF Tomkowicz B Perry WL Strohl WR. Adeno-Associated Virus (AAV) as a Vector for Gene Therapy. BioDrugs (2017) 31(4):317–34. doi: 10.1007/s40259-017-0234-5 PMC5548848 28669112

[8] Wu Z Asokan A Samulski RJ. Adeno-Associated Virus Serotypes: Vector Toolkit for Human Gene Therapy. Mol Ther (2006) 14(3):316–27. doi: 10.1016/j.ymthe.2006.05.009 16824801

[9] Mével M Bouzelha M Leray A Pacouret S Guilbaud M Penaud-Budloo M. Chemical Modification of the Adeno-Associated Virus Capsid to Improve Gene Delivery. Chem Sci (2020) 11(4):1122–31. doi: 10.1039/C9SC04189C PMC8145868 34084369

[10] Hinderer C Katz N Buza EL Dyer C Goode T Bell P. Severe Toxicity in Nonhuman Primates and Piglets Following High-Dose Intravenous Administration of an Adeno-Associated Virus Vector Expressing Human SMN. Hum Gene Ther (2018) 29(3):285–98. doi: 10.1089/hum.2018.015 PMC5865262 29378426

[11] Srivastava A. AAV Vectors: Are They Safe? Hum Gene Ther (2020) 31(13–14):697–9. doi: 10.1089/hum.2020.187 32611206

[12] Wilson JM Flotte TR. Moving Forward After Two Deaths in a Gene Therapy Trial of Myotubular Myopathy. Hum Gene Ther (2020) 31(13–14):695–6. doi: 10.1089/hum.2020.182 32605399

[13] Paulk N. Gene Therapy: It Is Time to Talk About High-Dose AAV: The Deaths of Two Children With X-Linked Myotubular Myopathy in the ASPIRO Trial Prompts a Reexamination of Vector Safety. Genet Eng Biotechnol News (2020) 40(9):14–6. doi: 10.1089/gen.40.09.04

[14] Calcedo R Chichester JA Wilson JM. Assessment of Humoral, Innate, and T-Cell Immune Responses to Adeno-Associated Virus Vectors. Hum Gene Ther Methods (2018) 29(2):86–95. doi: 10.1089/hgtb.2018.038 29668327

[15] Gao G Wang Q Calcedo R Mays L Bell P Wang L. Adeno-Associated Virus-Mediated Gene Transfer to Nonhuman Primate Liver Can Elicit Destructive Transgene-Specific T Cell Responses. Hum Gene Ther (2009) 20(9):930–42. doi: 10.1089/hum.2009.060 PMC2829282 19441963

[16] Mingozzi F High KA. Immune Responses to AAV Vectors: Overcoming Barriers to Successful Gene Therapy. Blood (2013) 122(1):23–36. doi: 10.1182/blood-2013-01-306647 23596044 PMC3701904

[17] Finn JD Hui D Downey HD Dunn D Pien GC Mingozzi F. Proteasome Inhibitors Decrease AAV2 Capsid Derived Peptide Epitope Presentation on MHC Class I Following Transduction. Mol Ther (2010) 18(1):135–42. doi: 10.1038/mt.2009.257 PMC2839204 19904235

[18] Pien GC Basner-Tschakarjan E Hui DJ Mentlik AN Finn JD Hasbrouck NC. Capsid Antigen Presentation Flags Human Hepatocytes for Destruction After Transduction by Adeno-Associated Viral Vectors. J Clin Invest (2009) 119(6):1688–95. doi: 10.1172/JCI36891 PMC2689109 19436115

[19] Mingozzi F Meulenberg JJ Hui DJ Basner-Tschakarjan E Hasbrouck NC Edmonson SA. AAV-1–Mediated Gene Transfer to Skeletal Muscle in Humans Results in Dose-Dependent Activation of Capsid-Specific T Cells. Blood (2009) 114(10):2077–86. doi: 10.1182/blood-2008-07-167510 PMC2744569 19506302

[20] Nathwani AC Tuddenham EGD Rangarajan S Rosales C McIntosh J Linch DC. Adenovirus-Associated Virus Vector–Mediated Gene Transfer in Hemophilia B. N Engl J Med (2011) 365(25):2357–65. doi: 10.1056/NEJMoa1108046 PMC3265081 22149959

[21] Herzog RW Cooper M Perrin GQ Biswas M Martino AT Morel L. Regulatory T Cells and TLR9 Activation Shape Antibody Formation to a Secreted Transgene Product in AAV Muscle Gene Transfer. Cell Immunol (2019) 342:103682. doi: 10.1016/j.cellimm.2017.07.012 28888664 PMC5794662

[22] Rogers GL Shirley JL Zolotukhin I Kumar SRP Sherman A Perrin GQ. Plasmacytoid and Conventional Dendritic Cells Cooperate in Crosspriming AAV Capsid-Specific CD8+ T Cells. Blood (2017) 129(24):3184–95. doi: 10.1182/blood-2016-11-751040 PMC5472899 28468798

[23] Rossi A Dupaty L Aillot L Zhang L Gallien C Hallek M. Vector Uncoating Limits Adeno-Associated Viral Vector-Mediated Transduction of Human Dendritic Cells and Vector Immunogenicity. Sci Rep (2019) 9(1):3631. doi: 10.1038/s41598-019-40071-1 30842485 PMC6403382

[24] Somanathan S Breous E Bell P Wilson JM. AAV Vectors Avoid Inflammatory Signals Necessary to Render Transduced Hepatocyte Targets for Destructive T Cells. Mol Ther (2010) 18(5):977–82. doi: 10.1038/mt.2010.40 PMC2890110 20234342

[25] Vandenberghe LH Wang L Somanathan S Zhi Y Figueredo J Calcedo R. Heparin Binding Directs Activation of T Cells Against Adeno-Associated Virus Serotype 2 Capsid. Nat Med (2006) 12(8):967–71. doi: 10.1038/nm1445 16845388

[26] Zhu J Huang X Yang Y. The TLR9-MyD88 Pathway Is Critical for Adaptive Immune Responses to Adenoassociated Virus Gene Therapy Vectors in Mice. J Clin Investig (2009) 119(8):2388–98. doi: 10.1172/JCI37607 PMC2719948 19587448

[27] Bartlett JS Kleinschmidt J Boucher RC Samulski RJ. Targeted Adeno-Associated Virus Vector Transduction of Nonpermissive Cells Mediated by a Bispecific F(Ab’γ) 2 Antibody. Nat Biotechnol (1999) 17(2):181–6. doi: 10.1038/6185 10052356

[28] Davidsson M Wang G Aldrin-Kirk P Cardoso T Nolbrant S Hartnor M. A Systematic Capsid Evolution Approach Performed *In Vivo* for the Design of AAV Vectors With Tailored Properties and Tropism. Proc Natl Acad Sci USA (2019) 116(52):27053–62. doi: 10.1073/pnas.1910061116 PMC6936499 31818949

[29] Davis AS Federici T Ray WC Boulis NM O’Connor D Clark KR. Rational Design and Engineering of a Modified Adeno-Associated Virus (AAV1)-Based Vector System for Enhanced Retrograde Gene Delivery. Neurosurgery (2015) 76(2):216–25. doi: 10.1227/NEU.0000000000000589 25549186

[30] Lee EJ Guenther CM Suh J. Adeno-Associated Virus (AAV) Vectors: Rational Design Strategies for Capsid Engineering. Curr Opin BioMed Eng (2018) 7:58–63. doi: 10.1016/j.cobme.2018.09.004 31106283 PMC6516759

[31] Sen D. Improving Clinical Efficacy of Adeno Associated Vectors by Rational Capsid Bioengineering. J BioMed Sci (2014) 21(1):103. doi: 10.1186/s12929-014-0103-1 25425174 PMC4251935

[32] Chan KY Jang MJ Yoo BB Greenbaum A Ravi N Wu W-L. Engineered AAVs for Efficient Noninvasive Gene Delivery to the Central and Peripheral Nervous Systems. Nat Neurosci (2017) 20(8):1172–9. doi: 10.1038/nn.4593 PMC5529245 28671695

[33] Dalkara D Byrne LC Klimczak RR Visel M Yin L Merigan WH. *In Vivo*-Directed Evolution of a New Adeno-Associated Virus for Therapeutic Outer Retinal Gene Delivery From the Vitreous. Sci Transl Med (2013) 5(189):189ra76. doi: 10.1126/scitranslmed.3005708 23761039

[34] Deverman BE Pravdo PL Simpson BP Kumar SR Chan KY Banerjee A. Cre-Dependent Selection Yields AAV Variants for Widespread Gene Transfer to the Adult Brain. Nat Biotechnol (2016) 34(2):204–9. doi: 10.1038/nbt.3440 PMC5088052 26829320

[35] Excoffon KJDA Koerber JT Dickey DD Murtha M Keshavjee S Kaspar BK. Directed Evolution of Adeno-Associated Virus to an Infectious Respiratory Virus. Proc Natl Acad Sci USA (2009) 106(10):3865–70. doi: 10.1073/pnas.0813365106 PMC2646629 19237554

[36] Grimm D Lee JS Wang L Desai T Akache B Storm TA. *In Vitro* and *In Vivo* Gene Therapy Vector Evolution via Multispecies Interbreeding and Retargeting of Adeno-Associated Viruses. J Virol (2008) 82(12):5887–911. doi: 10.1128/JVI.00254-08 PMC2395137 18400866

[37] Körbelin J Sieber T Michelfelder S Lunding L Spies E Hunger A. Pulmonary Targeting of Adeno-Associated Viral Vectors by Next-Generation Sequencing-Guided Screening of Random Capsid Displayed Peptide Libraries. Mol Ther (2016) 24(6):1050–61. doi: 10.1038/mt.2016.62 PMC4923327 27018516

[38] Kotterman MA Schaffer DV. Engineering Adeno-Associated Viruses for Clinical Gene Therapy. Nat Rev Genet (2014) 15(7):445–51. doi: 10.1038/nrg3742 PMC4393649 24840552

[39] Maheshri N Koerber JT Kaspar BK Schaffer DV. Directed Evolution of Adeno-Associated Virus Yields Enhanced Gene Delivery Vectors. Nat Biotechnol (2006) 24(2):198–204. doi: 10.1038/nbt1182 16429148

[40] Müller OJ Kaul F Weitzman MD Pasqualini R Arap W Kleinschmidt JA. Random Peptide Libraries Displayed on Adeno-Associated Virus to Select for Targeted Gene Therapy Vectors. Nat Biotechnol (2003) 21(9):1040–6. doi: 10.1038/nbt856 12897791

[41] Ogden PJ Kelsic ED Sinai S Church GM. Comprehensive AAV Capsid Fitness Landscape Reveals a Viral Gene and Enables Machine-Guided Design. Science (2019) 366(6469):1139–43. doi: 10.1126/science.aaw2900 PMC7197022 31780559

[42] Ojala DS Sun S Santiago-Ortiz JL Shapiro MG Romero PA Schaffer DV. *In Vivo* Selection of a Computationally Designed SCHEMA AAV Library Yields a Novel Variant for Infection of Adult Neural Stem Cells in the SVZ. Mol Ther (2018) 26(1):304–19. doi: 10.1016/j.ymthe.2017.09.006 PMC5762983 28988711

[43] Pekrun K De Alencastro G Luo Q-J Liu J Kim Y Nygaard S. Using a Barcoded AAV Capsid Library to Select for Clinically Relevant Gene Therapy Vectors. JCI Insight (2019) 4(22):e131610. doi: 10.1172/jci.insight.131610 PMC6948855 31723052

[44] Pulicherla N Shen S Yadav S Debbink K Govindasamy L Agbandje-McKenna M. Engineering Liver-Detargeted AAV9 Vectors for Cardiac and Musculoskeletal Gene Transfer. Mol Ther (2011) 19(6):1070–8. doi: 10.1038/mt.2011.22 PMC3129791 21364538

[45] Ravindra Kumar S Miles TF Chen X Brown D Dobreva T Huang Q. Multiplexed Cre-Dependent Selection Yields Systemic AAVs for Targeting Distinct Brain Cell Types. Nat Methods (2020) 17:541–50. doi: 10.1038/s41592-020-0799-7 PMC7219404 32313222

[46] Tervo DG Hwang BY Viswanathan S Gaj T Lavzin M Ritola KD. A Designer AAV Variant Permits Efficient Retrograde Access to Projection Neurons. Neuron (2016) 92(2):372–82. doi: 10.1016/j.neuron.2016.09.021 PMC5872824 27720486

[47] Ying Y Müller OJ Goehringer C Leuchs B Trepel M Katus HA. Heart-Targeted Adeno-Associated Viral Vectors Selected by *In Vivo* Biopanning of a Random Viral Display Peptide Library. Gene Ther (2010) 17(8):980–90. doi: 10.1038/gt.2010.44 20393510

[48] Arruda VR Fields PA Milner R Wainwright L De Miguel MP Donovan PJ. Lack of Germline Transmission of Vector Sequences Following Systemic Administration of Recombinant AAV-2 Vector in Males. Mol Ther (2001) 4(6):586–92. doi: 10.1006/mthe.2001.0491 11735343

[49] Deleage C Wietgrefe SW Del Prete G Morcock DR Hao XP Piatak M. Defining HIV and SIV Reservoirs in Lymphoid Tissues. Pathog Immun (2016) 1(1):68–106. doi: 10.20411/pai.v1i1.100 27430032 PMC4943335

[50] Deleage C Chan CN Busman-Sahay K Estes JD. Next-Generation *In Situ* Hybridization Approaches to Define and Quantify HIV and SIV Reservoirs in Tissue Microenvironments. Retrovirology (2018) 15(1):4. doi: 10.1186/s12977-017-0387-9 29316956 PMC5761108

[51] Grabinski TM Kneynsberg A Manfredsson FP Kanaan NM. A Method for Combining RNAscope *in Situ* Hybridization With Immunohistochemistry in Thick Free-Floating Brain Sections and Primary Neuronal Cultures. PloS One (2015) 10(3):e0120120. doi: 10.1371/journal.pone.0120120 25794171 PMC4368734

[52] Hunter JE Gurda BL Yoon SY Castle MJ Wolfe JH. *In Situ* Hybridization for Detection of AAV-Mediated Gene Expression. Methods Mol Biol (2019) 1950:107–22. doi: 10.1007/978-1-4939-9139-6_6 PMC7029785 30783970

[53] Miao CH Nakai H Thompson AR Storm TA Chiu W Snyder RO. Nonrandom Transduction of Recombinant Adeno-Associated Virus Vectors in Mouse Hepatocytes *In Vivo*: Cell Cycling Does Not Influence Hepatocyte Transduction. J Virol (2000) 74(8):3793–803. doi: 10.1128/JVI.74.8.3793-3803.2000 PMC111888 10729154

[54] Polinski NK Gombash SE Manfredsson FP Lipton JW Kemp CJ Cole-Strauss A. Recombinant Adenoassociated Virus 2/5-Mediated Gene Transfer Is Reduced in the Aged Rat Midbrain. Neurobiol Aging (2015) 36(2):1110–20. doi: 10.1016/j.neurobiolaging.2014.07.047 PMC4315740 25457558

[55] Polinski NK Manfredsson FP Benskey MJ Fischer DL Kemp CJ Steece-Collier K. Impact of Age and Vector Construct on Striatal and Nigral Transgene Expression. Mol Ther Methods Clin Dev (2016) 3:16082. doi: 10.1038/mtm.2016.82 27933309 PMC5142515

[56] Puray-Chavez M Tedbury PR Huber AD Ukah OB Yapo V Liu D. Multiplex Single-Cell Visualization of Nucleic Acids and Protein During HIV Infection. Nat Commun (2017) 8(1):1882. doi: 10.1038/s41467-017-01693-z 29192235 PMC5709414

[57] Wang SK Lapan SW Hong CM Krause TB Cepko CL. *In Situ* Detection of Adeno-Associated Viral Vector Genomes With SABER-FISH. Mol Ther Methods Clin Dev (2020) 19:376–86. doi: 10.1016/j.omtm.2020.10.003 PMC7658570 33209963

[58] Zhang X Lu W Zheng Y Wang W Bai L Chen L. *In Situ* Analysis of Intrahepatic Virological Events in Chronic Hepatitis B Virus Infection. J Clin Invest (2016) 126(3):1079–92. doi: 10.1172/JCI83339 PMC4767362 26901811

[59] Zhao J Yue Y Patel A Wasala L Karp JF Zhang K. High-Resolution Histological Landscape of AAV DNA Distribution in Cellular Compartments and Tissues Following Local and Systemic Injection. Mol Ther Methods Clin Dev (2020) 18:856–68. doi: 10.1016/j.omtm.2020.08.006 PMC7479330 32953935

[60] Liu Q Yang Y Fan X. Microvascular Pericytes in Brain-Associated Vascular Disease. Biomed Pharmacother (2020) 121:109633. doi: 10.1016/j.biopha.2019.109633 31743876

[61] Marques S Zeisel A Codeluppi S van Bruggen D Mendanha Falcao A Xiao L. Oligodendrocyte Heterogeneity in the Mouse Juvenile and Adult Central Nervous System. Science (2016) 352(6291):1326–9. doi: 10.1126/science.aaf6463 PMC5221728 27284195

[62] Berto S Liu Y Konopka G. Genomics at Cellular Resolution: Insights Into Cognitive Disorders and Their Evolution. Hum Mol Genet (2020) 29(R1):R1–9. doi: 10.1093/hmg/ddaa117 PMC7530518 32566943

[63] Gokce O Stanley GM Treutlein B Neff NF Camp JG Malenka RC. Cellular Taxonomy of the Mouse Striatum as Revealed by Single-Cell RNA-Seq. Cell Rep (2016) 16(4):1126–37. doi: 10.1016/j.celrep.2016.06.059 PMC5004635 27425622

[64] Tasic B Menon V Nguyen TN Kim TK Jarsky T Yao Z. Adult Mouse Cortical Cell Taxonomy Revealed by Single Cell Transcriptomics. Nat Neurosci (2016) 19(2):335–46. doi: 10.1038/nn.4216 PMC4985242 26727548

[65] Tasic B Yao Z Graybuck LT Smith KA Nguyen TN Bertagnolli D. Shared and Distinct Transcriptomic Cell Types Across Neocortical Areas. Nature (2018) 563(7729):72–8. doi: 10.1038/s41586-018-0654-5 PMC6456269 30382198

[66] Zeisel A Hochgerner H Lönnerberg P Johnsson A Memic F van der Zwan J. Molecular Architecture of the Mouse Nervous System. Cell (2018) 174(4):999–1014.e22. doi: 10.1016/j.cell.2018.06.021 30096314 PMC6086934

[67] Lähnemann D Köster J Szczurek E McCarthy DJ Hicks SC Robinson MD. Eleven Grand Challenges in Single-Cell Data Science. Genome Biol (2020) 21(1):31. doi: 10.1186/s13059-020-1926-6 32033589 PMC7007675

[68] Yang S Corbett SE Koga Y Wang Z Johnson WE Yajima M. Decontamination of Ambient RNA in Single-Cell RNA-Seq With DecontX. Genome Biol (2020) 21(1):57. doi: 10.1186/s13059-020-1950-6 32138770 PMC7059395

[69] Zheng GXY Terry JM Belgrader P Ryvkin P Bent ZW Wilson R. Massively Parallel Digital Transcriptional Profiling of Single Cells. Nat Commun (2017) 8(1):14049. doi: 10.1038/ncomms14049 28091601 PMC5241818

[70] Korsunsky I Millard N Fan J Slowikowski K Zhang F Wei K. Fast, Sensitive and Accurate Integration of Single-Cell Data With Harmony. Nat Methods (2019) 16(12):1289–96. doi: 10.1038/s41592-019-0619-0 PMC6884693 31740819

[71] Lin Y Ghazanfar S Wang KYX Gagnon-Bartsch JA Lo KK Su X. Scmerge Leverages Factor Analysis, Stable Expression, and Pseudoreplication to Merge Multiple Single-Cell RNA-Seq Datasets. Proc Natl Acad Sci USA (2019) 116(20):9775–84. doi: 10.1073/pnas.1820006116 PMC6525515 31028141

[72] Lopez R Regier J Cole MB Jordan MI Yosef N. Deep Generative Modeling for Single-Cell Transcriptomics. Nat Methods (2018) 15(12):1053–8. doi: 10.1038/s41592-018-0229-2 PMC6289068 30504886

[73] Lun ATL Riesenfeld S Andrews T Dao TP Gomes T Marioni JC. EmptyDrops: Distinguishing Cells From Empty Droplets in Droplet-Based Single-Cell RNA Sequencing Data. Genome Biol (2019) 20(1):63. doi: 10.1186/s13059-019-1662-y 30902100 PMC6431044

[74] Macosko EZ Basu A Satija R Nemesh J Shekhar K Goldman M. Highly Parallel Genome-Wide Expression Profiling of Individual Cells Using Nanoliter Droplets. Cell (2015) 161(5):1202–14. doi: 10.1016/j.cell.2015.05.002 PMC4481139 26000488

[75] Huang KW Sabatini BL. Single-Cell Analysis of Neuroinflammatory Responses Following Intracranial Injection of G-Deleted Rabies Viruses. Front Cell Neurosci (2020) 14:65. doi: 10.3389/fncel.2020.00065 32265666 PMC7098990

[76] Duan D. Systemic AAV Micro-Dystrophin Gene Therapy for Duchenne Muscular Dystrophy. Mol Ther (2018) 26(10):2337–56. doi: 10.1016/j.ymthe.2018.07.011 PMC6171037 30093306

[77] Hösel M Broxtermann M Janicki H Esser K Arzberger S Hartmann P. Toll-Like Receptor 2–Mediated Innate Immune Response in Human Nonparenchymal Liver Cells Toward Adeno-Associated Viral Vectors. Hepatology (2012) 55(1):287–97. doi: 10.1002/hep.24625 21898480

[78] Martino AT Suzuki M Markusic DM Zolotukhin I Ryals RC Moghimi B. The Genome of Self-Complementary Adeno-Associated Viral Vectors Increases Toll-Like Receptor 9–Dependent Innate Immune Responses in the Liver. Blood (2011) 117(24):6459–68. doi: 10.1182/blood-2010-10-314518 PMC3123017 21474674

[79] Shao W Earley LF Chai Z Chen X Sun J He T. Double-Stranded RNA Innate Immune Response Activation From Long-Term Adeno-Associated Virus Vector Transduction. JCI Insight (2018) 3(12):e120474. doi: 10.1172/jci.insight.120474 PMC6124417 29925692

[80] Zaiss AK Cotter MJ White LR Clark SA Wong NCW Holers VM. Complement Is an Essential Component of the Immune Response to Adeno-Associated Virus Vectors. J Virol (2008) 82(6):2727–40. doi: 10.1128/JVI.01990-07 PMC2259003 18199646

[81] George LA Sullivan SK Giermasz A Rasko JEJ Samelson-Jones BJ Ducore J. Hemophilia B Gene Therapy With a High-Specific-Activity Factor IX Variant. N Engl J Med (2017) 377(23):2215–27. doi: 10.1056/NEJMoa1708538 PMC6029626 29211678

[82] Manno CS Pierce GF Arruda VR Glader B Ragni M Rasko JJE. Successful Transduction of Liver in Hemophilia by AAV-Factor IX and Limitations Imposed by the Host Immune Response. Nat Med (2006) 12(3):342–7. doi: 10.1038/nm1358 16474400

[83] Mingozzi F Maus MV Hui DJ Sabatino DE Murphy SL Rasko JEJ. CD8 + T-Cell Responses to Adeno-Associated Virus Capsid in Humans. Nat Med (2007) 13(4):419–22. doi: 10.1038/nm1549 17369837

[84] Nathwani AC Reiss UM Tuddenham EGD Rosales C Chowdary P McIntosh J. Long-Term Safety and Efficacy of Factor IX Gene Therapy in Hemophilia B. N Engl J Med (2014) 371(21):1994–2004. doi: 10.1056/NEJMoa1407309 25409372 PMC4278802

[85] Colella P Ronzitti G Mingozzi F. Emerging Issues in AAV-Mediated *In Vivo* Gene Therapy. Mol Ther Methods Clin Dev (2018) 8:87–104. doi: 10.1016/j.omtm.2017.11.007 29326962 PMC5758940

[86] Shirley JL de Jong YP Terhorst C Herzog RW. Immune Responses to Viral Gene Therapy Vectors. Mol Ther (2020) 28(3):709–22. doi: 10.1016/j.ymthe.2020.01.001 PMC7054714 31968213

[87] Chamberlin NL Du B de Lacalle S Saper CB. Recombinant Adeno-Associated Virus Vector: Use for Transgene Expression and Anterograde Tract Tracing in the CNS. Brain Res (1998) 793(1–2):169–75. doi: 10.1016/S0006-8993(98)00169-3 PMC4961038 9630611

[88] McCown TJ Xiao X Li J Breese GR Jude Samulski R. Differential and Persistent Expression Patterns of CNS Gene Transfer by an Adeno-Associated Virus (AAV) Vector. Brain Res (1996) 713(1–2):99–107. doi: 10.1016/0006-8993(95)01488-8 8724980

[89] Lowenstein P Mandel R Xiong W Kroeger K Castro M. Immune Responses to Adenovirus and Adeno-Associated Vectors Used for Gene Therapy of Brain Diseases: The Role of Immunological Synapses in Understanding the Cell Biology of Neuroimmune Interactions. CGT (2007) 7(5):347–60. doi: 10.2174/156652307782151498 PMC2268649 17979681

[90] Flytzanis NC Goeden N Goertsen D Cummins A Pickel J Gradinaru V. Broad Gene Expression Throughout the Mouse and Marmoset Brain After Intravenous Delivery of Engineered AAV Capsids. bioRxiv (2020). 2020.06.16.152975. doi: 10.1101/2020.06.16.152975

[91] Chang H Lim J Ha M Kim VN. TAIL-Seq: Genome-Wide Determination of Poly(A) Tail Length and 3′ End Modifications. Mol Cell (2014) 53(6):1044–52. doi: 10.1016/j.molcel.2014.02.007 24582499

[92] Shin S Park J. Characterization of Sequence-Specific Errors in Various Next-Generation Sequencing Systems. Mol Biosyst (2016) 12(3):914–22. doi: 10.1039/C5MB00750J 26790373

[93] Wolock SL Lopez R Klein AM. Scrublet: Computational Identification of Cell Doublets in Single-Cell Transcriptomic Data. Cell Syst (2019) 8(4):281–91.e9. doi: 10.1016/j.cels.2018.11.005 30954476 PMC6625319

[94] Xu C Lopez R Mehlman E Regier J Jordan MI Yosef N. Probabilistic Harmonization and Annotation of Single-Cell Transcriptomics Data With Deep Generative Models. Mol Syst Biol (2021) 17(1):e9620. doi: 10.15252/msb.20209620 33491336 PMC7829634

[95] Yao Z van Velthoven CTJ Nguyen TN Goldy J Sedeno-Cortes AE Baftizadeh F. A Taxonomy of Transcriptomic Cell Types Across the Isocortex and Hippocampal Formation. Cell (2021) 184(12):3222–41.e26. doi: 10.1016/j.cell.2021.04.021 34004146 PMC8195859

[96] Cao Z-J Wei L Lu S Yang D-C Gao G. Searching Large-Scale scRNA-Seq Databases *via* Unbiased Cell Embedding With Cell BLAST. Nat Commun (2020) 11(1):3458. doi: 10.1038/s41467-020-17281-7 32651388 PMC7351785

[97] Tan Y Cahan P. SingleCellNet: A Computational Tool to Classify Single Cell RNA-Seq Data Across Platforms and Across Species. Cell Syst (2019) 9(2):207–13.e2. doi: 10.1016/j.cels.2019.06.004 31377170 PMC6715530

[98] Choi HMT Beck VA Pierce NA. Next-Generation *In Situ* Hybridization Chain Reaction: Higher Gain, Lower Cost, Greater Durability. ACS Nano (2014) 8(5):4284–94. doi: 10.1021/nn405717p PMC4046802 24712299

[99] Patriarchi T Cho JR Merten K Howe MW Marley A Xiong W-H. Ultrafast Neuronal Imaging of Dopamine Dynamics With Designed Genetically Encoded Sensors. Science (2018) 360(6396):eaat4422. doi: 10.1126/science.aat4422 29853555 PMC6287765

[100] He L Vanlandewijck M Raschperger E Andaloussi Mäe M Jung B Lebouvier T. Analysis of the Brain Mural Cell Transcriptome. Sci Rep (2016) 6(1):35108. doi: 10.1038/srep35108 27725773 PMC5057134

[101] Vanlandewijck M He L Mäe MA Andrae J Ando K Del Gaudio F. A Molecular Atlas of Cell Types and Zonation in the Brain Vasculature. Nature (2018) 554(7693):475–80. doi: 10.1038/nature25739 29443965

[102] Yang AC Vest RT Kern F Lee DP Maat CA Losada PM. A Human Brain Vascular Atlas Reveals Diverse Cell Mediators of Alzheimer’s Disease Risk. bioRxiv (2021) 2021.04.26.441262. doi: 10.1101/2021.04.26.441262 PMC9635042 35165441

[103] Ferreira MP Nagai MA. PHLDA3 (Pleckstrin Homology-Like Domain, Family A, Member 3). Atlas Genet Cytogenetics Oncol Haematol (2019) 24(1):8–12. doi: 10.4267/2042/70642

[104] Ghouzzi VE Bianchi FT Molineris I Mounce BC Berto GE Rak M. ZIKA Virus Elicits P53 Activation and Genotoxic Stress in Human Neural Progenitors Similar to Mutations Involved in Severe Forms of Genetic Microcephaly and P53. Cell Death Dis (2016) 7(10):e2440–0. doi: 10.1038/cddis.2016.266 PMC5133962 27787521

[105] Amirinejad R Rezaei M Shirvani-Farsani Z. An Update on Long Intergenic Noncoding RNA P21: A Regulatory Molecule With Various Significant Functions in Cancer. Cell Biosci (2020) 10(1):82. doi: 10.1186/s13578-020-00445-9 32582435 PMC7310005

[106] Lorenzon E Colladel R Andreuzzi E Marastoni S Todaro F Schiappacassi M. MULTIMERIN2 Impairs Tumor Angiogenesis and Growth by Interfering With VEGF-A/VEGFR2 Pathway. Oncogene (2012) 31(26):3136–47. doi: 10.1038/onc.2011.487 22020326

[107] Fischietti M Arslan AD Sassano A Saleiro D Majchrzak-Kita B Ebine K. Slfn2 Regulates Type I Interferon Responses by Modulating the NF-κb Pathway. Mol Cell Biol (2018) 38(16):e00053-18. doi: 10.1128/MCB.00053-18 29866656 PMC6066750

[108] Mathys H Adaikkan C Gao F Young JZ Manet E Hemberg M. Temporal Tracking of Microglia Activation in Neurodegeneration at Single-Cell Resolution. Cell Rep (2017) 21(2):366–80. doi: 10.1016/j.celrep.2017.09.039 PMC5642107 29020624

[109] Jordão MJC Sankowski R Brendecke SM Sagar Locatelli G Tai Y-H. Single-Cell Profiling Identifies Myeloid Cell Subsets With Distinct Fates During Neuroinflammation. Science (2019) 363(6425):eaat7554. doi: 10.1126/science.aat7554 30679343

[110] Gralinski LE Ashley SL Dixon SD Spindler KR. Mouse Adenovirus Type 1-Induced Breakdown of the Blood-Brain Barrier. JVI (2009) 83(18):9398–410. doi: 10.1128/JVI.00954-09 PMC2738240 19570856

[111] Chui R Dorovini-Zis K. Regulation of CCL2 and CCL3 Expression in Human Brain Endothelial Cells by Cytokines and Lipopolysaccharide. J Neuroinflamm (2010) 7(1):1. doi: 10.1186/1742-2094-7-1 PMC2819252 20047691

[112] Nishimura H Akiyama T Irei I Hamazaki S Sadahira Y. Cellular Localization of Sphingosine-1-Phosphate Receptor 1 Expression in the Human Central Nervous System. J Histochem Cytochem (2010) 58(9):847–56. doi: 10.1369/jhc.2010.956409 PMC2924800 20566754

[113] Körbelin J Dogbevia G Michelfelder S Ridder DA Hunger A Wenzel J. A Brain Microvasculature Endothelial Cell-Specific Viral Vector With the Potential to Treat Neurovascular and Neurological Diseases. EMBO Mol Med (2016) 8(6):609–25. doi: 10.15252/emmm.201506078 PMC4888852 27137490

[114] De Alencastro G Pekrun K Valdmanis P Tiffany M Xu J Kay MA. Tracking Adeno-Associated Virus Capsid Evolution by High-Throughput Sequencing. Hum Gene Ther (2020) 31(9–10):553–64. doi: 10.1089/hum.2019.339 PMC7232707 32024384

[115] Herrmann A-K Bender C Kienle E Grosse S El Andari J Botta J. A Robust and All-Inclusive Pipeline for Shuffling of Adeno-Associated Viruses. ACS Synth Biol (2019) 8(1):194–206. doi: 10.1021/acssynbio.8b00373 30513195

[116] Paulk NK Pekrun K Zhu E Nygaard S Li B Xu J. Bioengineered AAV Capsids With Combined High Human Liver Transduction *In Vivo* and Unique Humoral Seroreactivity. Mol Ther (2018) 26(1):289–303. doi: 10.1016/j.ymthe.2017.09.021 29055620 PMC5763027

[117] Adachi K Enoki T Kawano Y Veraz M Nakai H. Drawing a High-Resolution Functional Map of Adeno-Associated Virus Capsid by Massively Parallel Sequencing. Nat Commun (2014) 5(1):3075. doi: 10.1038/ncomms4075 24435020 PMC3941020

[118] Chen KH Boettiger AN Moffitt JR Wang S Zhuang X. Spatially Resolved, Highly Multiplexed RNA Profiling in Single Cells. Science (2015) 348(6233):aaa6090. doi: 10.1126/science.aaa6090 25858977 PMC4662681

[119] Femino AM Fay FS Fogarty K Singer RH. Visualization of Single RNA Transcripts *In Situ* . Science (1998) 280(5363):585–90. doi: 10.1126/science.280.5363.585 9554849

[120] Lubeck E Coskun AF Zhiyentayev T Ahmad M Cai L. Single-Cell *In Situ* RNA Profiling by Sequential Hybridization. Nat Methods (2014) 11(4):360–1. doi: 10.1038/nmeth.2892 PMC4085791 24681720

[121] Shah S Lubeck E Zhou W Cai L. *In Situ* Transcription Profiling of Single Cells Reveals Spatial Organization of Cells in the Mouse Hippocampus. Neuron (2016) 92(2):342–57. doi: 10.1016/j.neuron.2016.10.001 PMC5087994 27764670

[122] Shah S Lubeck E Schwarzkopf M He TF Greenbaum A Sohn CH. Single-Molecule RNA Detection at Depth by Hybridization Chain Reaction and Tissue Hydrogel Embedding and Clearing. Development (2016) 143(15):2862–7. doi: 10.1242/dev.138560 PMC5004914 27342713

[123] Ke R Mignardi M Pacureanu A Svedlund J Botling J Wählby C. *In Situ* Sequencing for RNA Analysis in Preserved Tissue and Cells. Nat Methods (2013) 10(9):857–60. doi: 10.1038/nmeth.2563 23852452

[124] Lee JH Daugharthy ER Scheiman J Kalhor R Yang JL Ferrante TC. Highly Multiplexed Subcellular RNA Sequencing *In Situ* . Science (2014) 343(6177):1360–3. doi: 10.1126/science.1250212 PMC4140943 24578530

[125] Wang X Allen WE Wright MA Sylwestrak EL Samusik N Vesuna S. Three-Dimensional Intact-Tissue Sequencing of Single-Cell Transcriptional States. Science (2018) 361(6400):eaat5691. doi: 10.1126/science.aat5691 29930089 PMC6339868

[126] Liao J Lu X Shao X Zhu L Fan X. Uncovering an Organ’s Molecular Architecture at Single-Cell Resolution by Spatially Resolved Transcriptomics. Trends Biotechnol (2020) 39(1):43–58. doi: 10.1016/j.tibtech.2020.05.006 32505359

[127] McGinnis CS Patterson DM Winkler J Conrad DN Hein MY Srivastava V. MULTI-Seq: Sample Multiplexing for Single-Cell RNA Sequencing Using Lipid-Tagged Indices. Nat Methods (2019) 16(7):619–26. doi: 10.1038/s41592-019-0433-8 PMC6837808 31209384

[128] Stoeckius M Zheng S Houck-Loomis B Hao S Yeung BZ Mauck WM. Cell Hashing With Barcoded Antibodies Enables Multiplexing and Doublet Detection for Single Cell Genomics. Genome Biol (2018) 19(1):224. doi: 10.1186/s13059-018-1603-1 30567574 PMC6300015

[129] Achim K Pettit J-B Saraiva LR Gavriouchkina D Larsson T Arendt D. High-Throughput Spatial Mapping of Single-Cell RNA-Seq Data to Tissue of Origin. Nat Biotechnol (2015) 33(5):503–9. doi: 10.1038/nbt.3209 25867922

[130] Durruthy-Durruthy R Gottlieb A Heller S. 3D Computational Reconstruction of Tissues With Hollow Spherical Morphologies Using Single-Cell Gene Expression Data. Nat Protoc (2015) 10(3):459–74. doi: 10.1038/nprot.2015.022 PMC4523134 25675210

[131] Halpern KB Shenhav R Matcovitch-Natan O Tóth B Lemze D Golan M. Single-Cell Spatial Reconstruction Reveals Global Division of Labour in the Mammalian Liver. Nature (2017) 542(7641):352–6. doi: 10.1038/nature21065 PMC5321580 28166538

[132] Nitzan M Karaiskos N Friedman N Rajewsky N. Gene Expression Cartography. Nature (2019) 576(7785):132–7. doi: 10.1038/s41586-019-1773-3 31748748

[133] Satija R Farrell JA Gennert D Schier AF Regev A. Spatial Reconstruction of Single-Cell Gene Expression Data. Nat Biotechnol (2015) 33(5):495–502. doi: 10.1038/nbt.3192 25867923 PMC4430369

[134] Stuart T Butler A Hoffman P Hafemeister C Papalexi E Mauck WM. Comprehensive Integration of Single-Cell Data. Cell (2019) 177(7):1888–902.e21. doi: 10.1016/j.cell.2019.05.031 31178118 PMC6687398

[135] Ruden JB Dugan LL Konradi C. Parvalbumin Interneuron Vulnerability and Brain Disorders. Neuropsychopharmacol (2021) 46(2):279–87. doi: 10.1038/s41386-020-0778-9 PMC7852528 32722660

[136] Mossner JM Batista-Brito R Pant R Cardin JA. Developmental Loss of MeCP2 From VIP Interneurons Impairs Cortical Function and Behavior. eLife (2020) 9:e55639. doi: 10.7554/eLife.55639 32343226 PMC7213975

[137] Batista AR King OD Reardon CP Davis C Shankaracharya Philip V. Ly6a Differential Expression in Blood–Brain Barrier Is Responsible for Strain Specific Central Nervous System Transduction Profile of AAV-PHP.B. Hum Gene Ther (2020) 31(1–2):90–102. doi: 10.1089/hum.2019.186 31696742

[138] Hordeaux J Yuan Y Clark PM Wang Q Martino RA Sims JJ. The GPI-Linked Protein LY6A Drives AAV-PHP.B Transport Across the Blood-Brain Barrier. Mol Ther (2019) 27(5):912–21. doi: 10.1016/j.ymthe.2019.02.013 PMC6520463 30819613

[139] Huang Q Chan KY Tobey IG Chan YA Poterba T Boutros CL. Delivering Genes Across the Blood-Brain Barrier: LY6A, a Novel Cellular Receptor for AAV-PHP.B Capsids. PloS One (2019) 14(11):e0225206. Di Pasquale G, editor. doi: 10.1371/journal.pone.0225206 31725765 PMC6855452

[140] Perez-Nievas BG Serrano-Pozo A. Deciphering the Astrocyte Reaction in Alzheimer’s Disease. Front Aging Neurosci (2018) 10:114. doi: 10.3389/fnagi.2018.00114 29922147 PMC5996928

[141] Wu YE Pan L Zuo Y Li X Hong W. Detecting Activated Cell Populations Using Single-Cell RNA-Seq. Neuron (2017) 96(2):313–29.e6. doi: 10.1016/j.neuron.2017.09.026 29024657

[142] Blanchard JW Bula M Davila-Velderrain J Akay LA Zhu L Frank A. Reconstruction of the Human Blood-Brain Barrier *In Vitro* Reveals a Pathogenic Mechanism of APOE4 in Pericytes. Nat Med (2020) 26(6):952–63. doi: 10.1038/s41591-020-0886-4 PMC7704032 32514169

[143] Montagne A Nation DA Sagare AP Barisano G Sweeney MD Chakhoyan A. APOE4 Leads to Blood–Brain Barrier Dysfunction Predicting Cognitive Decline. Nature (2020) 581(7806):71–6. doi: 10.1038/s41586-020-2247-3 PMC7250000 32376954

[144] Yang Y Vidensky S Jin L Jie C Lorenzini I Frankl M. Molecular Comparison of GLT1+ and ALDH1L1+ Astrocytes *In Vivo* in Astroglial Reporter Mice. Glia (2011) 59(2):200–7. doi: 10.1002/glia.21089 PMC3199134 21046559

[145] Zhang Z Ma Z Zou W Guo H Liu M Ma Y. The Appropriate Marker for Astrocytes: Comparing the Distribution and Expression of Three Astrocytic Markers in Different Mouse Cerebral Regions. BioMed Res Int (2019) 2019:1–15. doi: 10.1155/2019/9605265 PMC6613026 31341912

[146] Johansen J Tornøe J Møller A Johansen TE. Increased *In Vitro* and *In Vivo* Transgene Expression Levels Mediated Through Cis -Acting Elements: Cis Elements Increased *Ex Vivo* Gene Expression. J Gene Med (2003) 5(12):1080–9. doi: 10.1002/jgm.444 14661183

[147] Qin JY Zhang L Clift KL Hulur I Xiang AP Ren B-Z. Systematic Comparison of Constitutive Promoters and the Doxycycline-Inducible Promoter. PloS One (2010) 5(5):e10611. Hansen IA, editor. doi: 10.1371/journal.pone.0010611 20485554 PMC2868906

[148] Chuah MK Petrus I De Bleser P Le Guiner C Gernoux G Adjali O. Liver-Specific Transcriptional Modules Identified by Genome-Wide *In Silico* Analysis Enable Efficient Gene Therapy in Mice and Non-Human Primates. Mol Ther (2014) 22(9):1605–13. doi: 10.1038/mt.2014.114 PMC4435486 24954473

[149] Jüttner J Szabo A Gross-Scherf B Morikawa RK Rompani SB Hantz P. Targeting Neuronal and Glial Cell Types With Synthetic Promoter AAVs in Mice, Non-Human Primates and Humans. Nat Neurosci (2019) 22(8):1345–56. doi: 10.1038/s41593-019-0431-2 31285614

[150] Rincon MY Sarcar S Danso-Abeam D Keyaerts M Matrai J Samara-Kuko E. Genome-Wide Computational Analysis Reveals Cardiomyocyte-Specific Transcriptional Cis-Regulatory Motifs That Enable Efficient Cardiac Gene Therapy. Mol Ther (2015) 23(1):43–52. doi: 10.1038/mt.2014.178 25195597 PMC4426801

[151] Hrvatin S Tzeng CP Nagy MA Stroud H Koutsioumpa C Wilcox OF. A Scalable Platform for the Development of Cell-Type-Specific Viral Drivers. eLife (2019) 8:e48089. West AE, Dulac C, editors. doi: 10.7554/eLife.48089 31545165 PMC6776442

[152] Mich JK Graybuck LT Hess EE Mahoney JT Kojima Y Ding Y. Functional Enhancer Elements Drive Subclass-Selective Expression From Mouse to Primate Neocortex. Cell Rep (2020) 34(13):108754. doi: 10.1016/j.celrep.2021.108754 PMC8163032 33789096

[153] Gustafsson C Govindarajan S Minshull J. Codon Bias and Heterologous Protein Expression. Trends Biotechnol (2004) 22(7):346–53. doi: 10.1016/j.tibtech.2004.04.006 15245907

[154] Kodali MC Chen H Liao F-F. Temporal Unsnarling of Brain’s Acute Neuroinflammatory Transcriptional Profiles Reveals Panendothelitis as the Earliest Event Preceding Microgliosis. Mol Psychiatry (2020) s41380-020-00955-5. doi: 10.1038/s41380-020-00955-5 PMC7722246 33293688

[155] Zamagni A Pasini A Pirini F Ravaioli S Giordano E Tesei A. CDKN1A Upregulation and Cisplatin−Pemetrexed Resistance in Non−Small Cell Lung Cancer Cells. Int J Oncol (2020) 56(6):1574–84. doi: 10.3892/ijo.2020.5024 PMC7170038 32236605

[156] Helin E Matikainen S Julkunen I Heino J Hyypiä T Vainionpää R. Measles Virus Enhances the Expression of Cellular Immediate-Early Genes and DNA-Binding of Transcription Factor AP-1 in Lung Epithelial A549 Cells. Arch Virol (2002) 147(9):1721–32. doi: 10.1007/s00705-002-0835-1 12209312

[157] Chen W Zhang S Williams J Ju B Shaner B Easton J. A Comparison of Methods Accounting for Batch Effects in Differential Expression Analysis of UMI Count Based Single Cell RNA Sequencing. Comput Struct Biotechnol J (2020) 18:861–73. doi: 10.1016/j.csbj.2020.03.026 PMC7163294 32322368

[158] Ding J Adiconis X Simmons SK Kowalczyk MS Hession CC Marjanovic ND. Systematic Comparison of Single-Cell and Single-Nucleus RNA-Sequencing Methods. Nat Biotechnol (2020) 38(6):737–46. doi: 10.1038/s41587-020-0465-8 PMC7289686 32341560

[159] Lacar B Linker SB Jaeger BN Krishnaswami SR Barron JJ Kelder MJE. Nuclear RNA-Seq of Single Neurons Reveals Molecular Signatures of Activation. Nat Commun (2016) 7(1):11022. doi: 10.1038/ncomms11022 27090946 PMC4838832

[160] Rosenberg AB Roco CM Muscat RA Kuchina A Sample P Yao Z. Single-Cell Profiling of the Developing Mouse Brain and Spinal Cord With Split-Pool Barcoding. Science (2018) 360(6385):176–82. doi: 10.1126/science.aam8999 PMC7643870 29545511

[161] Challis RC Kumar SR Chan KY Challis C Beadle K Jang MJ. Systemic AAV Vectors for Widespread and Targeted Gene Delivery in Rodents. Nat Protoc (2019) 14:379–414. doi: 10.1038/s41596-018-0097-3 30626963 PMC13333184

[162] Pool A-H Wang T Stafford DA Chance RK Lee S Ngai J. The Cellular Basis of Distinct Thirst Modalities. Nature (2020) 588:112–7. doi: 10.1038/s41586-020-2821-8 PMC7718410 33057193

[163] Oikonomou G Altermatt M Zhang R Coughlin GM Montz C Gradinaru V. The Serotonergic Raphe Promote Sleep in Zebrafish and Mice. Neuron (2019) 103(4):686–701.e8. doi: 10.1016/j.neuron.2019.05.038 31248729 PMC6706304

[164] Schindelin J Arganda-Carreras I Frise E Kaynig V Longair M Pietzsch T. Fiji: An Open-Source Platform for Biological-Image Analysis. Nat Methods (2012) 9(7):676–82. doi: 10.1038/nmeth.2019 PMC3855844 22743772

[165] Love MI Huber W Anders S. Moderated Estimation of Fold Change and Dispersion for RNA-Seq Data With Deseq2. Genome Biol (2014) 15(12):550. doi: 10.1186/s13059-014-0550-8 25516281 PMC4302049

[166] Wolf FA Angerer P Theis FJ. SCANPY: Large-Scale Single-Cell Gene Expression Data Analysis. Genome Biol (2018) 19(1):15. doi: 10.1186/s13059-017-1382-0 29409532 PMC5802054

[167] Cahoy JD Emery B Kaushal A Foo LC Zamanian JL Christopherson KS. A Transcriptome Database for Astrocytes, Neurons, and Oligodendrocytes: A New Resource for Understanding Brain Development and Function. J Neurosci (2008) 28(1):264–78. doi: 10.1523/JNEUROSCI.4178-07.2008 PMC6671143 18171944

[168] Sun W Cornwell A Li J Peng S Osorio MJ Aalling N. SOX9 Is an Astrocyte-Specific Nuclear Marker in the Adult Brain Outside the Neurogenic Regions. J Neurosci (2017) 37(17):4493–507. doi: 10.1523/JNEUROSCI.3199-16.2017 PMC5413187 28336567

[169] Lin Y-S Wang H-Y Huang D-F Hsieh P-F Lin M-Y Chou C-H. Neuronal Splicing Regulator RBFOX3 (NeuN) Regulates Adult Hippocampal Neurogenesis and Synaptogenesis. PloS One (2016) 11(10):e0164164. Borlongan CV, editor. doi: 10.1371/journal.pone.0164164 27701470 PMC5049801

[170] Song HW Foreman KL Gastfriend BD Kuo JS Palecek SP Shusta EV. Transcriptomic Comparison of Human and Mouse Brain Microvessels. Sci Rep (2020) 10(1):12358. doi: 10.1038/s41598-020-69096-7 32704093 PMC7378255

[171] Veys K Fan Z Ghobrial M Bouché A García-Caballero M Vriens K. Role of the GLUT1 Glucose Transporter in Postnatal CNS Angiogenesis and Blood-Brain Barrier Integrity. Circ Res (2020) 127(4):466–82. doi: 10.1161/CIRCRESAHA.119.316463 PMC7386868 32404031

[172] Winkler EA Bell RD Zlokovic BV. Pericyte-Specific Expression of PDGF Beta Receptor in Mouse Models With Normal and Deficient PDGF Beta Receptor Signaling. Mol Neurodegeneration (2010) 5(1):32. doi: 10.1186/1750-1326-5-32 PMC2936891 20738866

[173] Capellera-Garcia S Pulecio J Dhulipala K Siva K Rayon-Estrada V Singbrant S. Defining the Minimal Factors Required for Erythropoiesis Through Direct Lineage Conversion. Cell Rep (2016) 15(11):2550–62. doi: 10.1016/j.celrep.2016.05.027 PMC4914771 27264182

[174] Chasseigneaux S Moraca Y Cochois-Guégan V Boulay A-C Gilbert A Le Crom S. Isolation and Differential Transcriptome of Vascular Smooth Muscle Cells and Mid-Capillary Pericytes From the Rat Brain. Sci Rep (2018) 8(1):12272. doi: 10.1038/s41598-018-30739-5 30116021 PMC6095852

[175] Dai J Bercury KK Ahrendsen JT Macklin WB. Olig1 Function Is Required for Oligodendrocyte Differentiation in the Mouse Brain. J Neurosci (2015) 35(10):4386–402. doi: 10.1523/JNEUROSCI.4962-14.2015 PMC4461695 25762682

[176] Suzuki N Sekimoto K Hayashi C Mabuchi Y Nakamura T Akazawa C. Differentiation of Oligodendrocyte Precursor Cells From Sox10-Venus Mice to Oligodendrocytes and Astrocytes. Sci Rep (2017) 7(1):14133. doi: 10.1038/s41598-017-14207-0 29074959 PMC5658394

[177] Miron VE Kuhlmann T Antel JP. Cells of the Oligodendroglial Lineage, Myelination, and Remyelination. Biochim Biophys Acta (BBA) - Mol Basis Dis (2011) 1812(2):184–93. doi: 10.1016/j.bbadis.2010.09.010 20887785

