# Supplementary material for: Deep Parallel Characterization of AAV Tropism and AAV-Mediated Transcriptional Changes via Single-Cell RNA Sequencing
Source: Front Immunol. 2021 Oct 21;12:730825. doi: 10.3389/fimmu.2021.730825 (PMC8574206; doi:10.3389/fimmu.2021.730825)
Supplement: Supplementary file 1 [file DataSheet_1.pdf]

frontiers

Supplementary Material

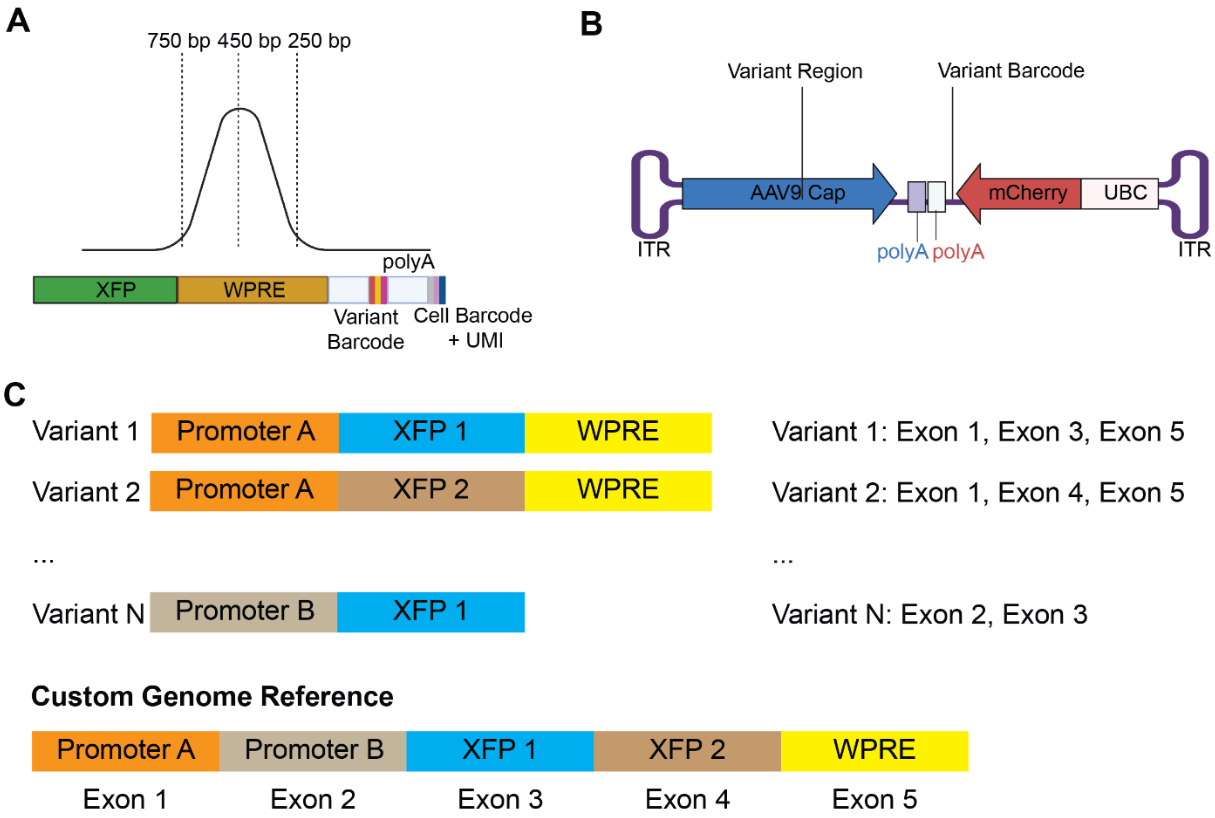

Supplemental Figure 1. Plasmid details. *(A) Size of typical transcriptome cDNA library post-fragmentation. Both distinguishing XFPs and variant barcodes fall outside the typical capture region of single-cell RNA sequencing workflows. (B) UBC-mCherry-AAV-cap-in-cis plasmid used for 7-variant barcoded pool. (C) Visualization of the construction procedure for the custom genome reference. Variant cargos are segmented into common and uncommon regions, and each unique segment is concatenated together as a contiguous gene. Variants are defined as different splicings of the custom AAV gene.*

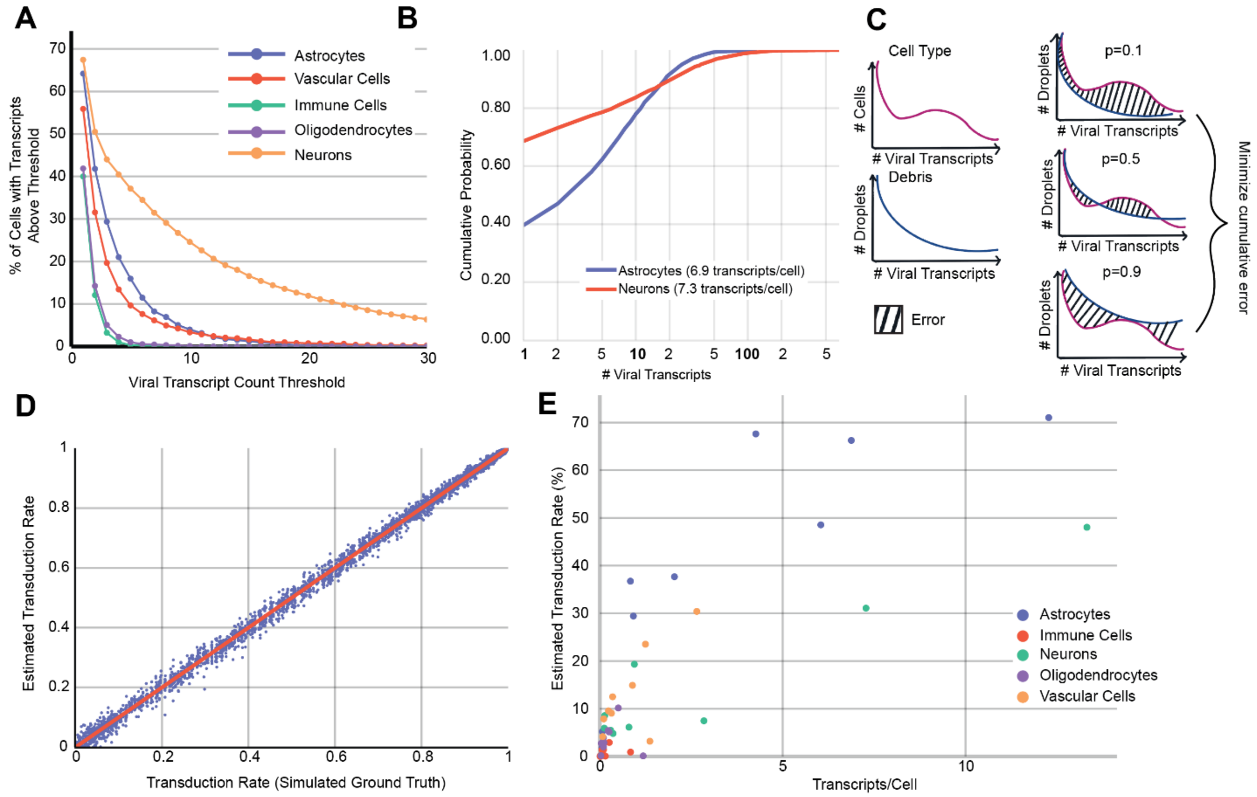

Supplemental Figure 2. Expression rate estimation. *(A) Percent of cells expressing AAV-PHP.eB cargo transcripts above a fixed threshold in a single sample. (B) An example of the distribution of viral transcript counts in a single animal from AAV-PHP.eB carrying CAG-mNeonGreen-WPRE in neurons and astrocytes. (C) Visualization of our expression-rate estimation algorithm. The distribution of the cell type of interest and background debris is obtained. An error is calculated for different estimates of the percent of the cells that express background levels of transcripts. This error is minimized to find the best fit. (D) Performance of the expression rate estimation algorithm on simulated data consisting of negative binomial distributions with parameters r between 0.1 and 10 and p between 0.001 and 0.99, spaced evenly apart. (E) Comparison between mean transcripts/cell (x) and the estimated transduction rate (y) in major cell types for AAV-PHP.eB across 9 samples.*

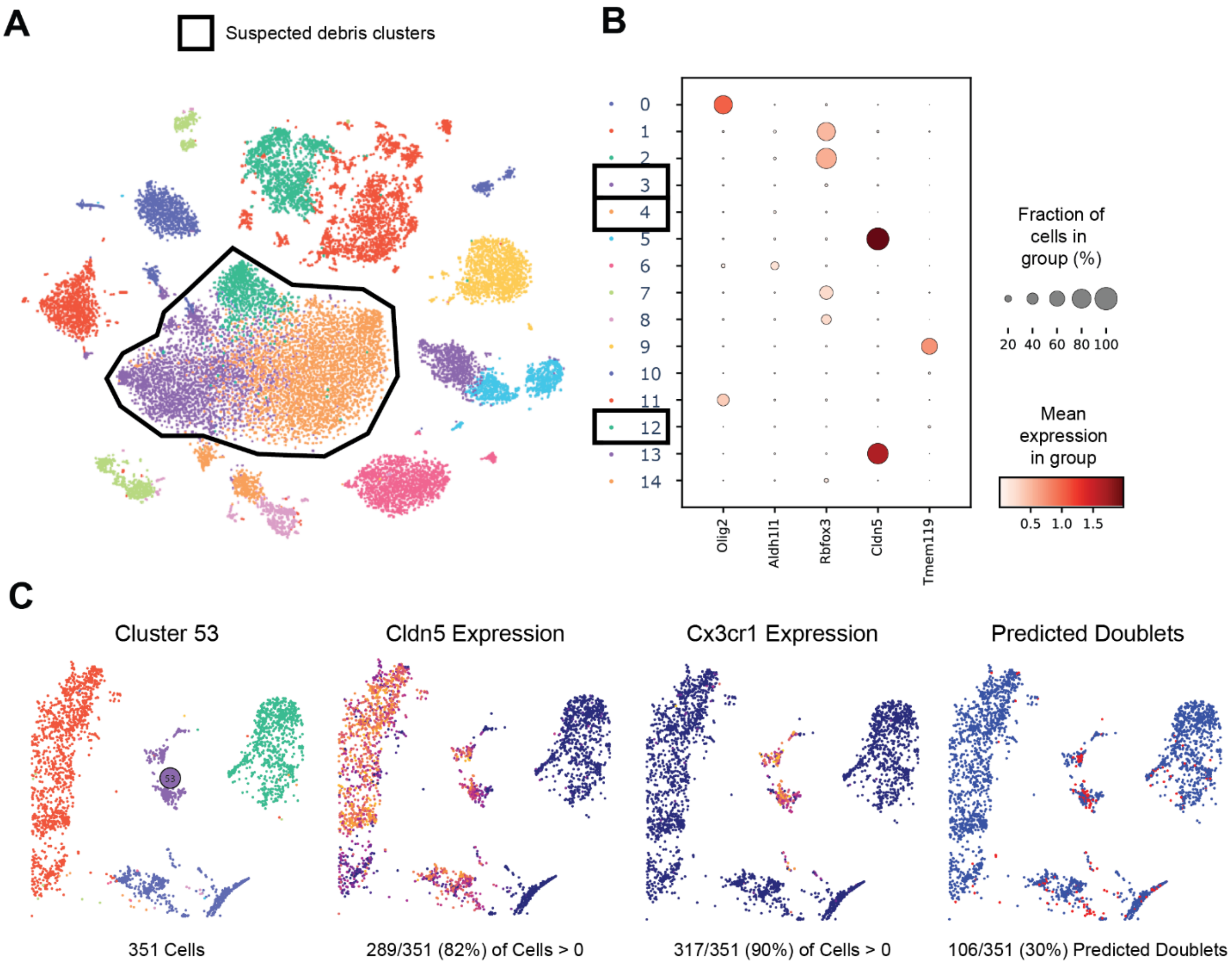

Supplemental Figure 3: Noise from debris and doublets. *(A) An example of a Cell Ranger filtered dataset. This is a t-SNE projection of the log-normalized gene expression space. Suspected debris clusters are outlined. (B) Marker gene expression for the major cell types in the brain—Oligodendrocytes/Olig2, Astrocytes/Aldh1l1, Neurons/Rbfox3, Vascular Cells/Cldn5, Immune Cells/Tmem119—for each cluster. Darker colors indicate higher mean expression, and dot size correlates with the abundance of the gene in that cluster. (C) An example of a multiplet cluster from the joint scVI space of all training samples, projected via t-SNE. Cluster 51 is annotated, and raw gene expression of Cldn5 and Cx3cr1 are shown. The percentage of cells in cluster 51 expressing each marker gene is displayed. (right) Predicted doublets from Scrublet are overlaid in red.*

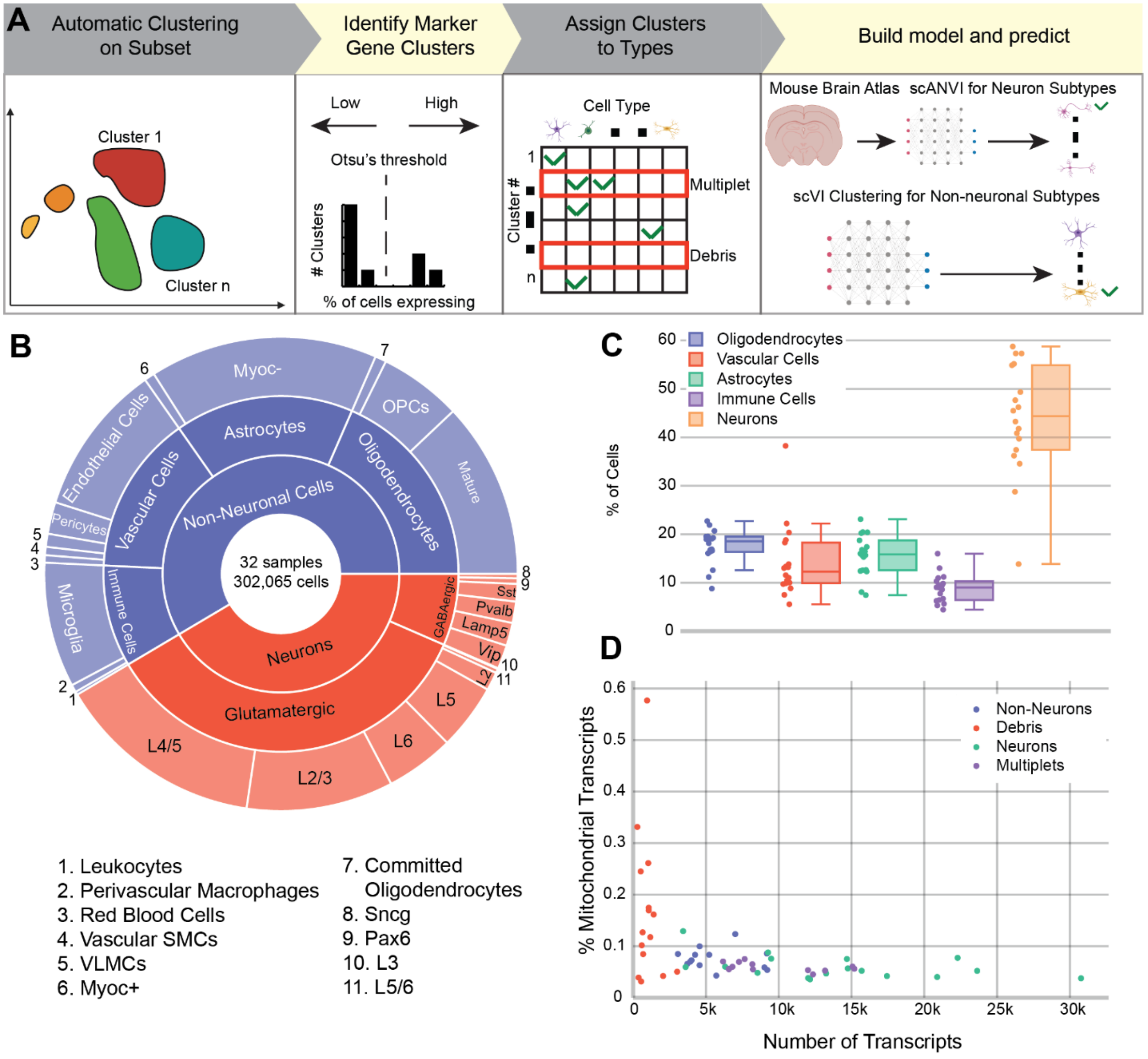

Supplemental Figure 4. Cell typing. *(A) Cell typing workflow. A subset of cells are used for training. For each marker gene, clusters expressing that marker gene are identified. Clusters that have no marker genes (debris) or are determined to be multiplets via Scrublet are marked for removal. Training data used to train a scANVI model to predict the remaining cells. A reference database can be used instead of manually labeled cells, as we did for neuronal subtypes. (B) Cell-type distribution of all labeled cells from our combined cell-type taxonomy. This includes samples described in the study as well as additional controls and animals used for troubleshooting and prototyping. (C) Cell-type percentages across the major cell types in the ten samples used for AAV tropism characterization. One of the samples, BC1, had dramatically fewer neurons than any other sample and correspondingly higher percentages of non-neurons. (D) Mitochondrial gene ratio and total transcript counts of the major cell type clusters in the ten samples used for tropism characterization.*

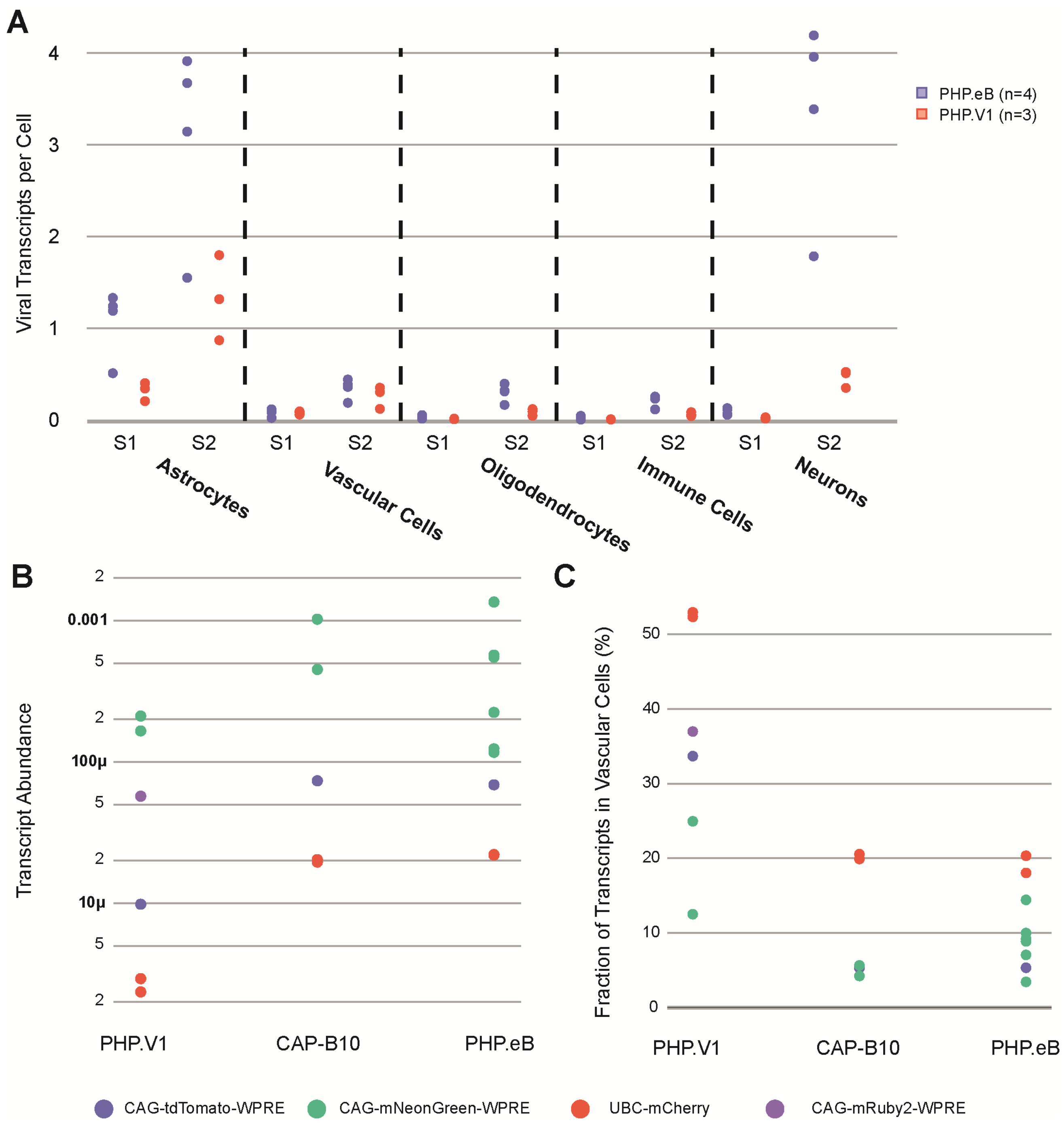

Supplemental Figure 5. Transcript expression. *(A) Viral transcript expression of different barcodes across two samples (S1, S2). Each point is a distinct barcode. (B) Viral transcript abundance in entire samples (viral transcripts / total transcripts) across different variants carrying different cargo. (C) Fraction of transcripts detected in vascular cells vs all other cell types.*

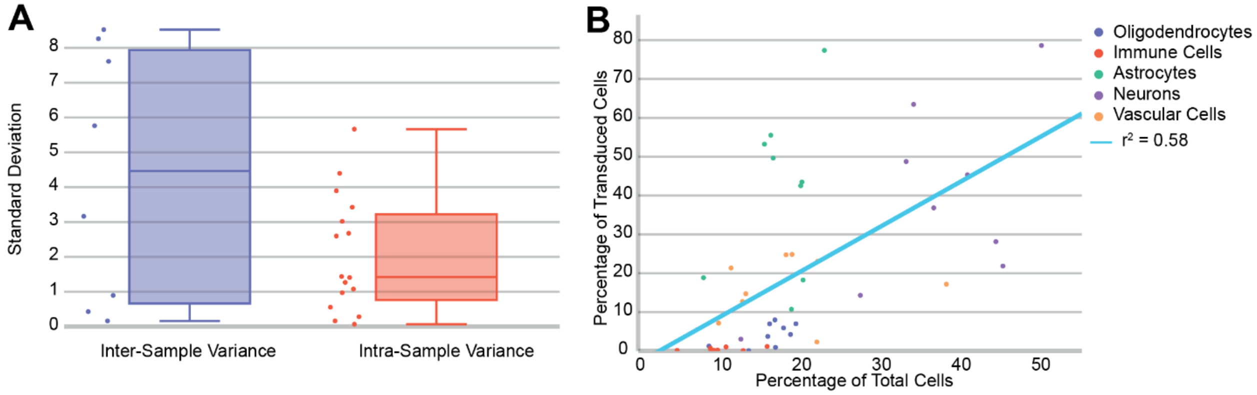

Supplemental Figure 6. Inter-sample variability. *(A) The standard deviations between measurements of the fraction of transduced cells in all major non-neuronal cell types in AAV-PHP.V1 and AAV-PHP.eB. Inter-sample variance (left) refers to the standard deviation between animals, and intra-sample variance (right) refers to the standard deviation between barcodes within the same animal. (B) The distribution of recovered cell types compared to the distribution of transduced cells across nine samples injected with AAV-PHP.eB.*

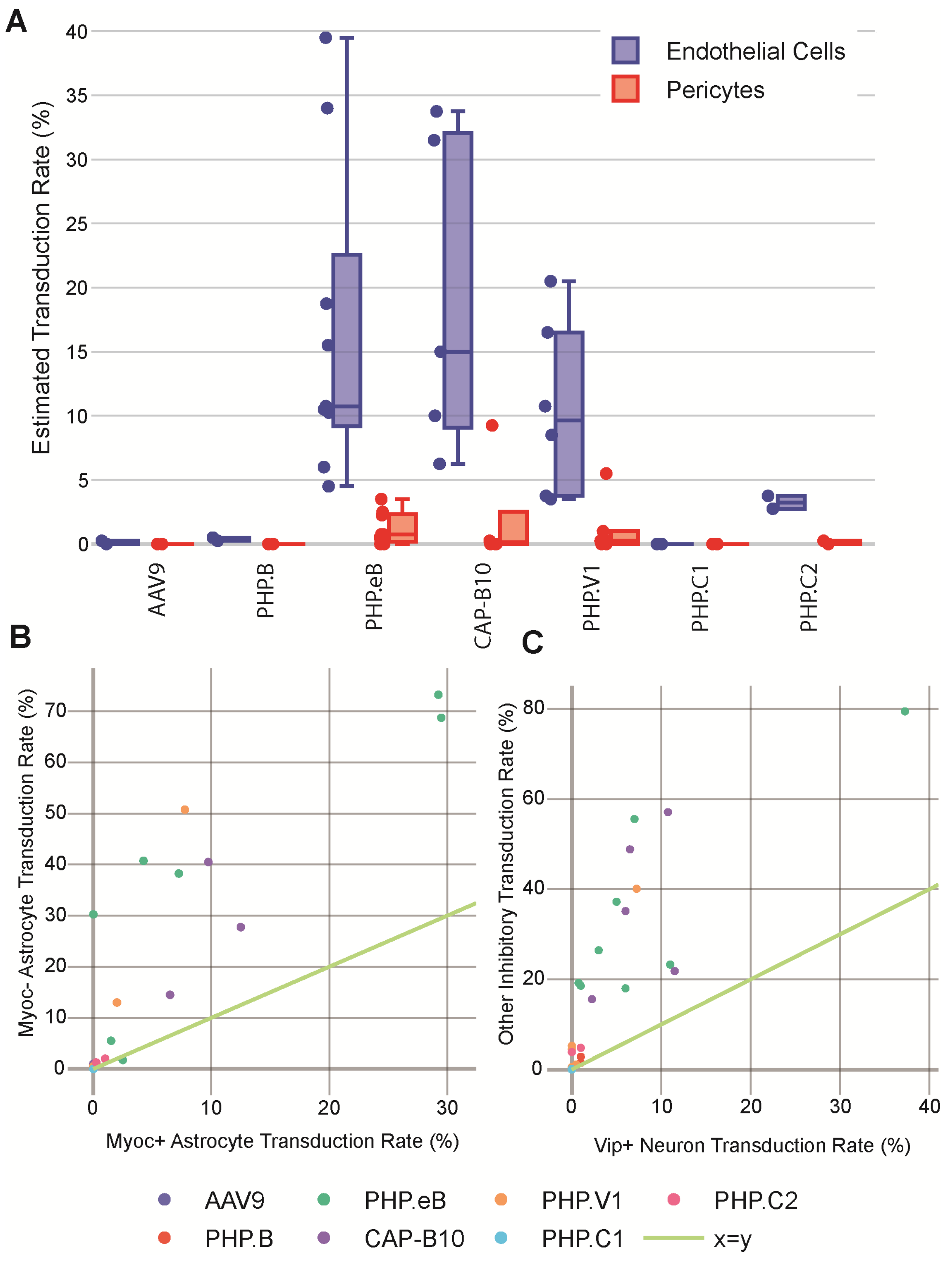

Supplemental Figure 7. Cell subtype inspection. *(A) Estimated transduction rate of endothelial cells vs pericytes across all samples and variants. (B) Pairwise transduction rate of Myoc+ and Myoc-*

astrocytes across all variants and samples. Each point is a single variant in a different sample. (C) Pairwise transduction rate of Vip+ neurons vs all other inhibitory neurons across all variants and samples.

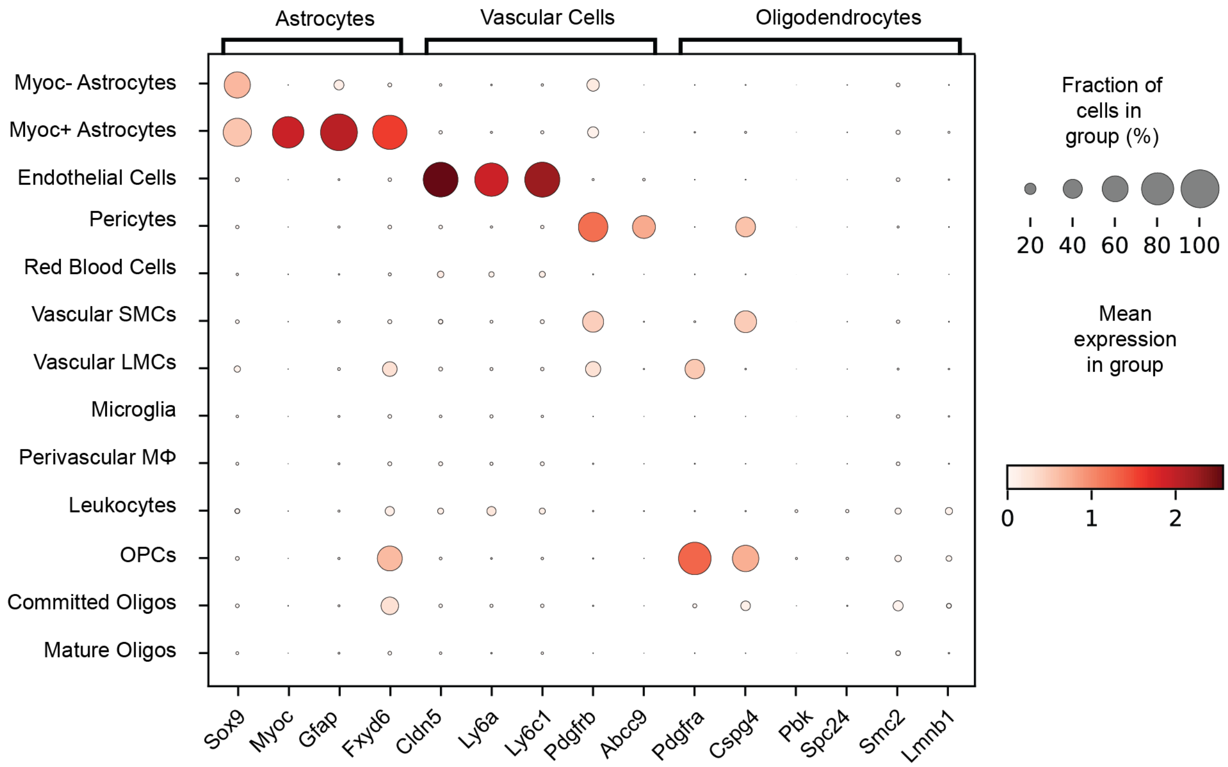

Supplemental Figure 8. Cell subtype markers. *Gene expression of additional marker genes for astrocyte, vascular and oligodendrocyte subtypes.*

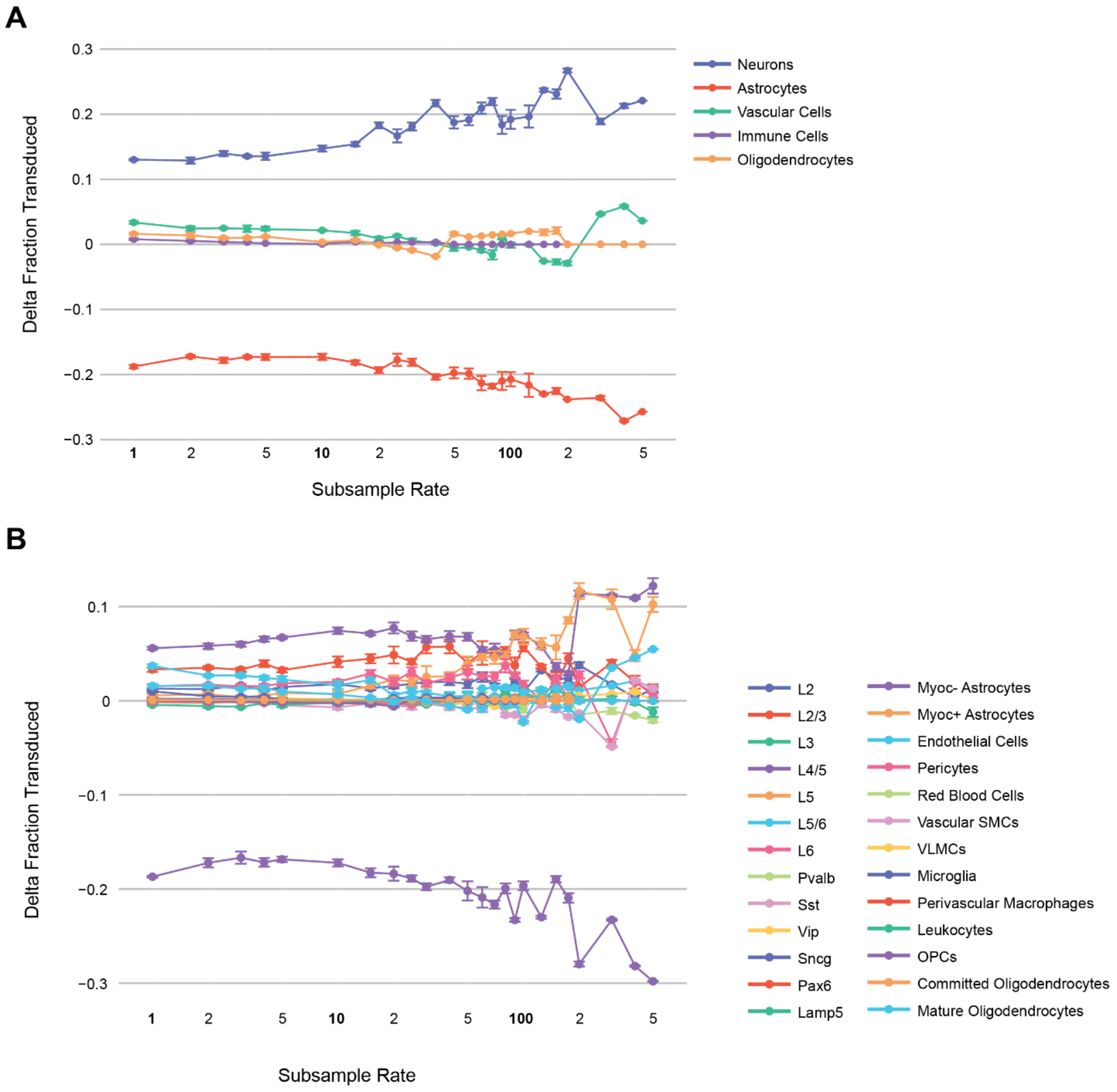

Supplemental Figure 9. Simulated scaling. *Delta fraction transduced between AAV.CAP-B10 and AAV-PHP.eB within a single animal at different rates of subsampled viral transcripts. Error bars indicate standard error over 5 simulations.*
