# Supplementary material for: Deep Parallel Characterization of AAV Tropism and AAV-Mediated Transcriptional Changes via Single-Cell RNA Sequencing
Source: Front Immunol. 2021 Oct 21;12:730825. doi: 10.3389/fimmu.2021.730825 (PMC8574206; doi:10.3389/fimmu.2021.730825)
Supplement: Supplementary file 2 [file DataSheet_2.pdf]

Supplemental Tables

Supplemental Table 1. Primers. *Primers used for round 1 and round 2 amplification of viral transcripts. Primers with TC1 and TC2 in the amplicon name indicate they were used only for those samples.*

**Amplicon**	**Read**	**Round**	**Sequence (Ns indicate Illumina sample index)**
**All Viruses**	1	1	CTACACGACGCTCTTCCGATCT
**All Viruses**	1	2	AATGATACGGCGACCACCGAGATCTACACTCTTTCC CTACACGACGCTCTTCCGAT
**mNeonGreen TC1**	2	1	TTCAAGGAGTGGCAAAAGGCCTTTACCGATGTGAT
**mRuby2**	2	1	CAACGGGAACATGCAGTTGCCAAGTTTGCTGG
**mNeonGreen**	2	1	TAACTATCTGAAGAACCAGCCGATGTAC
**tdTomato TC2**	2	1	AGGACTACACAATTGTCGAACAGTATGAG
**tdTomato**	2	1	ACAACGAGGACTACACCATCGTGG
**mCherry**	2	1	CATCGTGGAACAGTACGAACG
**WPRE**	2	2	CAAGCAGAAGACGGCATACGAGATNNNNNNNNGT GACTGGAGTTCAGACGTGTGCTCTTCCGATCTAGAC GAGTCGGATCTCCCT
**mNeonGreen**	2	2	CAAGCAGAAGACGGCATACGAGATNNNNNNNNGT GACTGGAGTTCAGACGTGTGCTCTTCCGATCTTTCA AGGAGTGGCAAAAGGC
**mRuby2**	2	2	CAAGCAGAAGACGGCATACGAGATNNNNNNNNGT GACTGGAGTTCAGACGTGTGCTCTTCCGATCTCAAC GGGAACATGCAGTTGC
**tdTomato TC2**	2	2	CAAGCAGAAGACGGCATACGAGATNNNNNNNNGT GACTGGAGTTCAGACGTGTGCTCTTCCGATCTGCAT GGACGAGCTGTACAAG
**tdTomato**	2	2	CAAGCAGAAGACGGCATACGAGATNNNNNNNNGT GACTGGAGTTCAGACGTGTGCTCTTCCGATCTCCTC TTTCTCTATGGGATGGATGA

**mCherry**	2	2	CAAGCAGAAGACGGCATACGAGATNNNNNNNNGTGACTGGAGTTCAGACGTGTGCTCTTCCGATCTCGGCATGGACGAGCTGT

Supplemental Table 2. Marker Genes.

**Cell Type**	**Marker Gene(s)**
**Astrocytes**	Aldh1l1 (168), Sox9 (169)
**Neurons**	Rbfox3 (170)
**Vascular Cells**	Cldn5 (171)
**Endothelial Cells**	Slc2a1 (172)
**Pericytes**	Pdgfrb (173), Rgs5, Abcc9 (100)
**Red Blood Cells**	Hba-a1, Hba-a2 (174)
**Vascular SMCs**	Acta2, Myh11, Tagln (175)
**Vascular LMCs**	Fam180a, Slc6a13, Dcn, Ptgds (61)
**Microglia**	Cx3cr1, Tmem119 (109)
**Leukocytes**	Itgal, Gzma (75)
**Perivascular Macropages**	Mrc1 (109)
**Oligodendrocytes**	Olig2 (176)
**OPCs**	Pdgfra, Cspg4 (177)
**Mature Oligos**	Mog, Mbp (178)
**Committed Oligos**	Ptprz1, Bmp4, Nkx2-2, Vcan (61)

Supplemental Table 3. scVI Hyperparameter Tuning

**Dispersion**	**Latent Lib Size**	**# Latent**	**# Layers**	**# Hidden**	**Test KL Divergence**
**Gene**	False	10	1	128	5366.4

**Gene-batch**	True	10	1	128	5406.1
**Gene**	True	10	1	128	5391.0
**Gene-batch**	True	50	2	512	5362.6
**Gene-batch**	False	10	1	128	5378.7
**Gene**	False	25	1	128	5354.6
**Gene**	False	25	2	256	5337.8
**Gene**	False	20	2	256	5336.7
**Gene**	False	40	4	1024	5338.0

Supplemental Table 4. Sample Metadata. *Supplemental file contains the following fields.*

Field Name	***Description***
**10X Version**	Whether the sample was processed using 10X V2 or V3 chemistry
**Animal ID**	A unique animal identifier. Some animals provided multiple samples
**Target # Cells**	The target number of cells for extraction. 1.6X this number is loaded into the 10X Chromium instrument
**Recovered Cells**	The number of cells recovered, after debris and multiplet filtering
**Typed Cells**	The number of cells typed after neuronal and non-neuronal cell assignment
**Cell Ranger # Cells**	The number of cells as predicted by Cell Ranger
**Predicted Multiplets**	The number of predicted multiplets
**Transcriptome Sequencing Depth**	The number of reads
**Transcriptome Reads/Cell**	The number of reads divided by the number of recovered cells
**Median UMIs/Cell**	Of the recovered cells, the median total UMI count

**Median Genes/Cell**	Of the recovered cells, the median number of genes detected with at least one transcript
**Variants Recovered**	Which variants were recovered from this sample. Samples labeled "Cell Typing Only" were not used for tropism or immune analysis, but were included in the cell type classifier
**Virus Sequencing Depth**	The number of reads of the amplified viral transcripts across all templates
**Virus Reads/Cell**	The read depth of the amplified viral transcripts
**Age at Extraction (Days)**	The age of the animal at extraction time
**Virus Incubation Time (Days)**	How many days prior to extraction the animal was injected
**Percent of Virus UMIs Determined**	What percent of transcriptome reads that aligned to the virus gene were disambiguated from the amplified lookup table

Supplemental Table 5. Variant Barcodes

**Variant**	**Cargo**	**Barcodes**
**AAV-PHP.eB**	pAAV:CAG-NLS-mNeonGreen	
**AAV-PHP.V1**	pAAV:CAG-NLS-mRuby2	
**AAV-PHP.eB**	pAAV:CAG-NLS-mNeonGreen	CCTGACA, GGACAGA, GCACAGA, CGAGAGA
**AAV-PHP.V1**	pAAV:CAG-tdTomato	
**AAV-PHP.V1**	pAAV:CAG-NLS-mNeonGreen	CAGTGTC, GAGAGTG, GTGTGAG
**AAV-CAP-B10**	pAAV:CAG-NLS-mNeonGreen	
**AAV-PHP.eB**	pAAV:CAG-NLS-tdTomato	
**AAV-CAP-B10**	pAAV:CAG-NLS-tdTomato	

**AAV9**	UBC-mCherry-AAV-cap-in-cis	CGTCTCAGCTATAACTTCCAA<br>CGAGGTCGTAAGGTCGGCATT<br>TGATTATCATGCCTGCTCAGG
**AAV-PHP.B**	UBC-mCherry-AAV-cap-in-cis	TATACCCAACCACTCAGTCCC<br>CGGTTTTAGCACGGCCATAGA<br>AAGCGATGTCTCTACACGATA
**AAV-PHP.eB**	UBC-mCherry-AAV-cap-in-cis	TACAGCTTTTTGACTGGAGGT<br>CTGGCATTAATACGCGGGTCA<br>TACAGGTCCTAGACAGGTGAT
**AAV-CAP-B10**	UBC-mCherry-AAV-cap-in-cis	GCTGGGCGTTAAAGTACTCGC<br>GCAACTGGGATAATCGTAGTC<br>AACGGAGTGAACGGACCCTAG
**AAV-PHP.V1**	UBC-mCherry-AAV-cap-in-cis	GTGGCGGGTTTCCGAAAAAGT<br>TCGTCGGCACTCTCTTAGAGC<br>CATGTGATAGTGAAGCACGCC
**AAV-PHP.C1**	UBC-mCherry-AAV-cap-in-cis	TCTGTGCTGCTCTTCTAACAA<br>TCTGACGGCGGGTAAACACTG<br>TGGCCACCCGCAGAGTATACT
**AAV-PHP.C2**	UBC-mCherry-AAV-cap-in-cis	GACTAGGGTAAGTGAGCTATG<br>CGAATTTCTTCCATACCTCCT<br>TAGTGCCAACAACGGAGAAGA

Supplemental Table 6. Cell Metadata. *Supplemental file contains one row per cell, with the following fields.*

Field Name	Description
**Cell ID**	The cell barcode plus a sample index
**Cell Type**	The final cell type of this cell
**AAV**	The number of UMIs in this cell that align to the custom AAV reference gene

**CCN202105041**	The predicted output of this cell from the droplet classifier – neurons vs non-neurons
**p_CCN202105041**	The probability of classification of this droplet type
**CCN202105050**	The predicted neuronal subtype of this cell, if it is a neuron
**p_CCN202105050**	The probability of classification of this neuron subtype
**CCN202105051**	The named non-neuronal subtype of this cell, if it is a non-neuron
**c_CCN202105051**	The cluster this cell was assigned to in the non-neuronal model
**X_CCN202105051**	The x-coordinate of a t-SNE projection of this cell in the non-neuronal model
**Y_CCN202105051**	The y-coordinate of a t-SNE projection of this cell in the non-neuronal model
**CCN202105070**	The cluster assignment of this cell in the joint neuronal and non-neuronal model
**X_CCN202105070**	The x-coordinate of a t-SNE projection of this cell in the joint neuronal and non-neuronal model
**Y_CCN202105070**	The y-coordinate of a t-SNE projection of this cell in the joint neuronal and non-neuronal model
**Cell Set**	The origin cell set of this cell
**[Variant]**	The number of UMIs in this cell that are associated with the given barcoded AAV variant

Supplemental Table 7. Transduction Rates by Cell Type. *Supplemental file contains one row per unique variant in each cell set.*

**Field Name**	**Description**
**Entry Id**	{Cell Set}-{Variant}
**Cell Set**	The source cell set
**Virus**	Which variant this row describes. Note: "AAV" means the transduction rate of any AAV present in the sample, as determined by a read's alignment to the

	custom AAV gene. All other variants originate from disambiguated counts.
**[Cell Type] Transduction Rate**	The estimated transduction rate of this variant in this cell type
**[Cell Type] Num Cells**	The number of total cells of this type in this cell set
**Field Name**	Description

Supplemental Table 8. Differentially Expressed Genes Across Time Points. *Supplemental file contains one tab per cell type, with the following fields.*

**Field Name**	**Description**
**Gene ID**	The Ensembl Gene ID
**Gene name**	The canonical gene name
**Mean expression**	The mean expression of this gene in this group
**L2FC**	The log fold change of this gene
**L2FC SE**	The standard error of the L2FC
**Stat**	The stat, as reported by DESeq2
**P-value**	The unadjusted P-value
**Adjusted P-value**	The adjusted P-value

References

169. Sun W, Cornwell A, Li J, Peng S, Osorio MJ, Aalling N, et al. SOX9 Is an Astrocyte-Specific Nuclear Marker in the Adult Brain Outside the Neurogenic Regions. J Neurosci. 2017 Apr 26;37(17):4493–507.

170. Lin Y-S, Wang H-Y, Huang D-F, Hsieh P-F, Lin M-Y, Chou C-H, et al. Neuronal Splicing Regulator RBFOX3 (NeuN) Regulates Adult Hippocampal Neurogenesis and Synaptogenesis. Borlongan CV, editor. PLoS ONE. 2016 Oct 4;11(10):e0164164.

171. Song HW, Foreman KL, Gastfriend BD, Kuo JS, Palecek SP, Shusta EV. Transcriptomic comparison of human and mouse brain microvessels. Sci Rep. 2020 Dec;10(1):12358.

172. Veys K, Fan Z, Ghobrial M, Bouché A, García-Caballero M, Vriens K, et al. Role of the GLUT1 Glucose Transporter in Postnatal CNS Angiogenesis and Blood-Brain Barrier Integrity. Circ Res. 2020 Jul 31;127(4):466–82.

173. Winkler EA, Bell RD, Zlokovic BV. Pericyte-specific expression of PDGF beta receptor in mouse models with normal and deficient PDGF beta receptor signaling. Mol Neurodegeneration. 2010;5(1):32.

174. Capellera-Garcia S, Pulecio J, Dhulipala K, Siva K, Rayon-Estrada V, Singbrant S, et al. Defining the Minimal Factors Required for Erythropoiesis through Direct Lineage Conversion. Cell Reports. 2016 Jun;15(11):2550–62.

175. Chasseigneaux S, Moraca Y, Cochois-Guégan V, Boulay A-C, Gilbert A, Le Crom S, et al. Isolation and differential transcriptome of vascular smooth muscle cells and mid-capillary pericytes from the rat brain. Sci Rep. 2018 Dec;8(1):12272.

176. Dai J, Bercury KK, Ahrendsen JT, Macklin WB. Olig1 Function Is Required for Oligodendrocyte Differentiation in the Mouse Brain. Journal of Neuroscience. 2015 Mar 11;35(10):4386–402.

177. Suzuki N, Sekimoto K, Hayashi C, Mabuchi Y, Nakamura T, Akazawa C. Differentiation of Oligodendrocyte Precursor Cells from Sox10-Venus Mice to Oligodendrocytes and Astrocytes. Sci Rep. 2017 Dec;7(1):14133.

178. Miron VE, Kuhlmann T, Antel JP. Cells of the oligodendroglial lineage, myelination, and remyelination. Biochimica et Biophysica Acta (BBA) - Molecular Basis of Disease. 2011 Feb;1812(2):184–93.
